# Gene expression during the first 28 days of axolotl limb regeneration I: Experimental design and global analysis of gene expression

**DOI:** 10.1002/reg2.37

**Published:** 2015-06-19

**Authors:** S. Randal Voss, Alex Palumbo, Radha Nagarajan, David M. Gardiner, Ken Muneoka, Arnold J. Stromberg, Antony T. Athippozhy

**Affiliations:** ^1^ Department of BiologyUniversity of KentuckyLexingtonKentucky40506USA; ^2^ Spinal Cord and Brain Injury Research CenterUniversity of KentuckyLexingtonKentucky40506USA; ^3^ Department of BiostatisticsUniversity of KentuckyLexingtonKentucky40506USA; ^4^ Department of Developmental and Cell BiologyUC‐IrvineIrvineCalifornia92697USA; ^5^ Department of Molecular and Cellular BiologyTulane UniversityNew OrleansLouisiana70118USA; ^6^ Department of StatisticsUniversity of KentuckyLexingtonKentucky40506USA

**Keywords:** Axolotl, limb, microarray, regeneration, transcription

## Abstract

While it is appreciated that global gene expression analyses can provide novel insights about complex biological processes, experiments are generally insufficiently powered to achieve this goal. Here we report the results of a robust microarray experiment of axolotl forelimb regeneration. At each of 20 post‐amputation time points, we estimated gene expression for 10 replicate RNA samples that were isolated from 1 mm of heterogeneous tissue collected from the distal limb tip. We show that the limb transcription program diverges progressively with time from the non‐injured state, and divergence among time adjacent samples is mostly gradual. However, punctuated episodes of transcription were identified for five intervals of time, with four of these coinciding with well‐described stages of limb regeneration—amputation, early bud, late bud, and pallet. The results suggest that regeneration is highly temporally structured and regulated by mechanisms that function within narrow windows of time to coordinate transcription within and across cell types of the regenerating limb. Our results provide an integrative framework for hypothesis generation using this complex and highly informative data set.

## Introduction

Among tetrapod vertebrates, only salamanders maintain potential throughout life to regenerate limbs after injury. This potential traces largely to signaling pathways and programs of development that are orchestrated by surviving cells near the site of injury. From the time of amputation until the successful reformation of a limb weeks later, these surviving cells display a diversity of behaviors, including death, growth, dedifferentiation, adhesion, communication, migration, mitosis, extracellular matrix remodeling, and differentiation (Carlson 2007). Changes in cellular behaviors during limb regeneration correlate with changes in transcription (Geraudie & Ferretti 1998). Various methods have been used to investigate spatial and temporal patterns of transcription during limb regeneration and identify candidate genes that are differentially expressed between regeneration‐competent and regeneration‐incompetent tissues (Monaghan et al. 2009, 2012; Campbell et al. 2011; Knapp et al. 2013; Stewart et al. 2013; Wu et al. 2013). Microarray and RNA‐Seq methodologies offer the potential to detail regeneration globally and in its entirety, as a dynamic continuous process that involves thousands of expression differences and hundreds of biological processes. However, to date, these powerful approaches have been used in experiments with sparse tissue sampling and no or few biological replicates.

**Figure 1:**
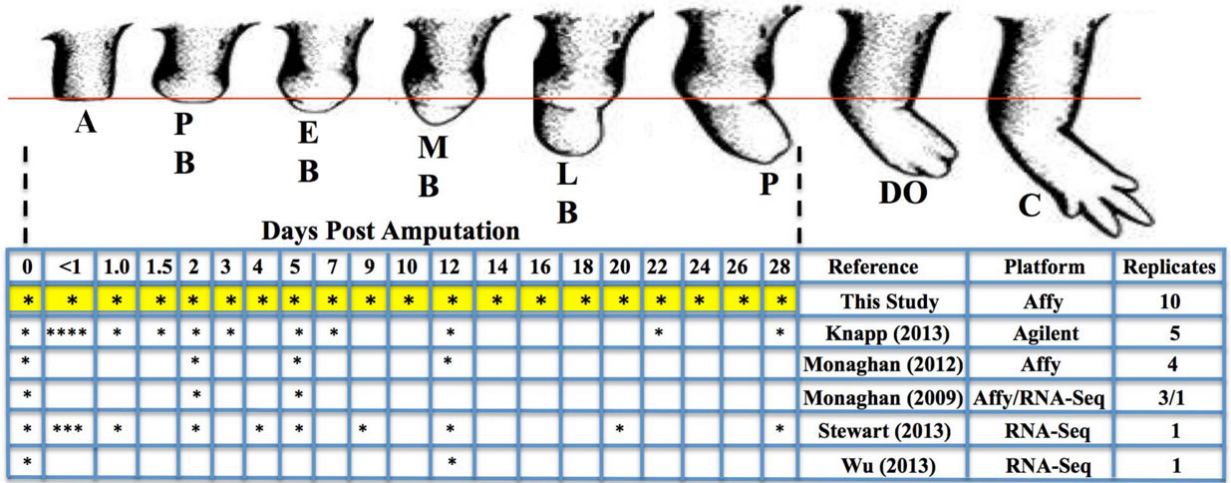
Experimental design compared to published studies that have examined gene expression during axolotl limb regeneration. The stages of limb regeneration are amputation (A), pre‐bud (PB), early bud (EB), medium bud (MB), late bud (LB), palette (P), digital outgrowth (DO), and completed (C). The vertical dashed lines show the stages that were examined and the red horizontal line shows the plane of amputation for an upper arm transection. The asterisks indicate when samples were collected for each experiment.

Here we present results from the largest microarray analysis of salamander limb regeneration performed to date (Fig. 1). We used 200 custom Affymetrix GeneChips to achieve 10× biological replication of tissues collected at 20 forelimb amputation and post‐amputation time points. In this contribution, we introduce the experiment and describe how transcription changed during the first 28 days of regeneration, focusing on global patterns of change. We identify intervals of time where transcriptional patterns changed the most, establishing these as focal points for subsequent analyses that relate expression patterns to biological processes and gene functions. An important objective of our work is to develop informational resources that show how changes in gene expression correlate with morphological, developmental, physiological, and molecular events that are well documented to occur during limb regeneration. Accordingly, the approximately 4,000,000 estimates of gene expression and 20,000 expression profiles from this experiment can be searched and viewed at Sal‐Site (www.ambystoma.org).

### Background

Before describing transcriptional data and results, we introduce details of limb regeneration and the experiment that was performed. This prelude provides context for interpreting gene expression changes, including information about limb regeneration from the literature and details about experimental design.

**Figure 2:**
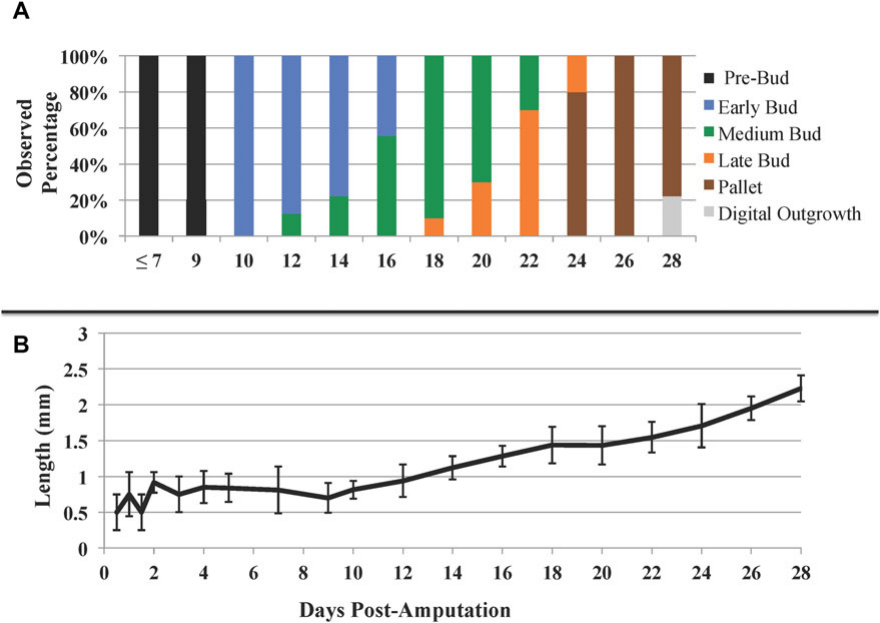
(A) How individuals were classified into developmental stages during the experiment. All individuals were classified as pre‐bud before day 10. (B) The length of the forelimb as measured distally from the initial plane of amputation. The error bars are standard deviations of the mean.

#### Limb regeneration

Limb regeneration is a temporal process that is often thought to entail a series of distinct developmental stages. Tank et al. (1976) provided an integrated histological and morphological staging scheme that focused on the blastema and limb outgrowth (Fig. 2). The blastema is a collection of mesenchymal‐like cells that organize beneath the wound epidermis approximately 6−12 days after limb amputation, the time depending upon a salamander's size and age. As the number of blastema cells increases, the limb bud lengthens and changes in shape. Prior to blastema formation there are essentially no external morphological criteria to reliably define distinct stages of limb regeneration. However, many biological processes are operative below the surface. Immediately after limb amputation, hemostatic mechanisms prevent blood loss and the wound surface is modified to facilitate the migration of cells from the dermis of the wound margin. Re‐epithelialization is complete within the first 10 h post‐amputation (Endo et al. 2004). Additional responses typical of epidermal wound healing are activated after amputation, including inflammation and extracellular matrix remodeling. However, equating epidermal wound healing with events that occur during the pre‐bud phase is a misnomer for several reasons. A unique wound epidermis covers an amputated limb; it differs structurally and functionally from the type of epidermis that covers a superficial skin wound (reviewed in Stocum 1995). Another key difference between epidermal wound healing and regeneration is that the latter encompasses repair of a greater diversity of tissues. In addition to epidermis, peripheral nerves, muscle, cartilage, connective tissue, and bone are repaired during regeneration. Thus, we call the period extending from limb amputation to the early bud stage as the pre‐bud (PB) phase of limb regeneration, and use subsequent developmental stages defined by Tank et al. (1976) as a working model to integrate transcriptional data and results. These later stages of regeneration are characterized by limb bud formation and outgrowth, which are known to be associated with blastema cell proliferation (Maden 1976). During early (EB) and medium bud (MB) stages, blastema cells stream distally to form an organized mass under a wound epithelium that has a distinct basal cell layer. Osteoclast activity peaks and then subsides during these stages as bone remodeling gives way to articular cartilage deposition. During the late bud (LB) stage, muscle and cartilage progenitors form condensations and cells begin to differentiate into mature cell types. Distinguishing between EB, MB, and LB at the morphological level rests on quantitative assessment of limb bud outgrowth. Dichotomizing a continuous growth process into discrete stages is difficult and explains why individuals in this study, that varied in body size and were obtained from different genetic crosses (spawns), were classified among EB, MB, and LB stages at a given post‐amputation sample point. The correspondence between developmental stage and chronological time was higher when individuals transitioned to morphologically distinct stages, when for example the limb showed initial outgrowth (EB) and digit primordial first appeared (pallet, P).

#### Experimental design

Our experimental design (Fig. 1) provided relatively deep sampling of the early regeneration program—eight time points were sampled from the time of amputation through day 5. More samples were collected during the first 5 days because we suspected that gene expression changes might be more dynamic during this period. During this period immune and inflammatory responses are deployed by resident and infiltrating cells from the blood stream, cells are lost as a result of necrosis and apoptosis, and cells synthesize transcripts consistent with cellular reprogramming and mitosis (Monaghan et al. 2009, 2012; Knapp et al. 2013). After day 5, we naively thought later stages of regeneration would be less dynamic and collected tissues at days 7, 9, 10, 12, and then every 2 days until we exhausted our resources (day 28). The 1‐day sampling interval between days 9 and 10 was designed to capture the transition point between the PB and EB stages. A similar temporal sampling scheme was used recently in two functional genomic studies of limb regeneration; however, one of these studies did not create biological replicate samples and the other did not sample as broadly or deeply. We collected 10 biological replicates for 20 different time points of regeneration, and did this after performing two different amputations along the forelimb—forearm and upper arm. Here, we represent the results of the forearm amputation; differences between forearm and upper arm amputation will be described in a separate paper. Future studies will be needed to detail gene expression during the terminal phase of limb regeneration.

#### Tissue

We collected 1.0 mm of heterogeneous tissue from the tips of amputated *Ambystoma mexicanum* limbs, isolated RNA, and performed microarray analysis using an Affymetrix GeneChip (Huggins et al. 2012). Thus our samples contained a heterogeneous mixture of tissue, including epidermis, wound epidermis, muscle, bone/cartilage, axons and nerve sheaths, blood vessels, connective tissues, fat, extracellular matrix, and blastema. There are at least four explanations for transcript abundance changes across a temporal series of heterogeneous tissue samples. First, cells are lost through cell death. After limb amputation, the injury environment is transformed by tissue histolysis, necrosis, and removal of tissue and cellular debris. This includes a dramatic, progressive reduction in transcript abundance for genes associated with muscle structure and function (Monaghan et al. 2009; Knapp et al. 2013). This correlates with muscle tissue histolysis and possibly changes in transcription by surviving muscle cells. Second, some cells increase their numerical abundance in samples through transport systems, cell migration, and proliferation. Early hemostatic responses are associated with the infiltration of nucleated red blood cells, platelets, macrophages, neutrophils, and other immunological cell types that are not well described in salamanders (but see Seifert et al. 2012; Godwin et al. 2013). The transcripts that these cells synthesize during the first couple of days contribute greatly to overall gene expression. As another example, later during regeneration when blastema cells proliferate and thus contribute more cells to samples, the mRNAs they synthesize bias samples for cell cycle transcripts (Monaghan et al. 2009; Knapp et al. 2013). Third, regenerative processes may alter transcription within surviving cells. Models of limb regeneration predict that stem‐cell‐like progenitors contribute to the blastema (e.g., McCusker and Gardiner 2013). Almost certainly, the transcripts that these cells synthesize must change over time as they become activated, migrate, proliferate, and differentiate. Fourth, the mode of tissue collection causes variability in the proportion of cell types represented among samples. For example, many researchers who perform limb regeneration experiments trim bones after amputation to facilitate wound closure. We did not introduce this surgical source of variation into our experimental design. As a result, our 1.0 mm sample of tissue contained proportionally more bone‐associated tissues than would be obtained had bones been trimmed at the time of amputation. We also note that during limb regeneration there is bud outgrowth. Thus, our later samples contained a greater proportion of newly regenerated tissue than early samples, which contained more tissue proximal to the amputation plane (i.e., stump tissue).

#### Data analysis

**Figure 3:**
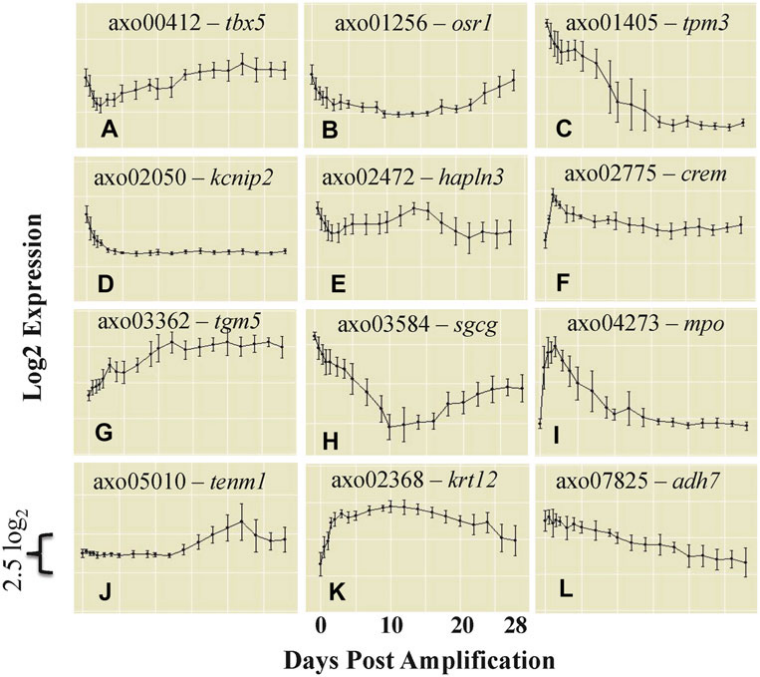
Sample gene expression profiles that can be accessed via Sal‐Site. Each estimate of transcript abundance was calculated from 10 replicate samples. The error bars are standard deviations.

There are many different ways to analyze gene expression data. We have used a variety of approaches in previous studies to examine gene expression change as a function of time, including *t* tests, multifactor ANOVA, repeated measures ANOVA, parametric and Bayesian linear and nonlinear modeling, piecewise regression, and clustering (e.g., Huggins et al. 2012; Li et al. 2004; Liu et al. 2005; Page et al. 2007, 2008, 2009, 2010, 2013; Monaghan et al. 2007, 2009, 2012; Athippozhy et al. 2014). These approaches are proven to be effective within the context of experimental designs that sample relatively few points in time. In exploratory analyses of the data from this study, where gene expression was estimated at 20 time points, we observed highly variable and complex patterns of gene expression change, with genes showing a diversity of linear and nonlinear patterns across few or many time points (Fig. 3). Also, the variance in mean expression across time varied within and among genes. We could not satisfactorily reduce the complexity of the overall data set using clustering and unbiased criteria to identify gene clusters (Dunn 1974; Rousseeuw 1987). As our objective in this first analysis was to detail global and local changes in gene expression, we reasoned that *t* tests could be used effectively to show the divergence of the limb regeneration program over time from the non‐amputated state, and to identify critical intervals of transcriptional regulation. Additional analyses of the overall data set, which is available from Sal‐Site (www.ambystoma.org) and GEO (GSE67118), will almost certainly extract additional useful information about the biology of limb regeneration.

## Results

### Developmental staging of samples

Each amputated limb from which tissue was collected for RNA isolation and microarray analysis was staged according to Tank et al. (1976). This allowed estimates of gene expression to be related to post‐amputation time points, gross morphology, and descriptions of histological change (Tank et al. 1976). The PB stage spanned the first 10 days, during which no limb outgrowth was observed (Fig. 2). At 10 days post‐amputation (DPA), all forelimb amputations presented characteristics of a symmetrical, contoured EB. Cone‐shaped MB limbs were observed from 12 to 22 DPA. LB limbs were evident as flattened cones and were observed from 18 to 24 DPA, and broad and flat palette (P) limbs were observed from 24 to 28 DPA. Two out of nine limbs at 28 DPA had four visible digit primordia characteristic of the digital outgrowth stage of regeneration. These results detail less developmental stage variation among individuals as they transitioned between PB and EB (i.e., bud formation), and MB and P. This suggests that post‐amputation time is highly correlated with discernible morphological changes, even changes that occur 28 days after amputation. In contrast, considerably more temporal variation was observed among individuals as they transitioned between EB, MB, and LB stages. In part, this probably reflects ambiguity in ascribing continuous variation in limb shape to discrete developmental stages, highlighting the need to identify gene expression changes that can better resolve cryptic regeneration processes.

**Figure 4:**
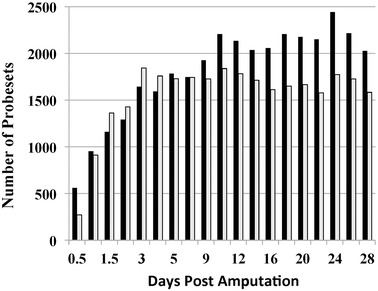
The number of probesets identified as significantly different on comparing post‐amputation samples to the day 0 sample. Bars show probesets with increasing (black bars) and decreasing (grey bars) changes in abundance.

### Total number of differently expressed genes as a function of time

To explore global transcriptional complexity of forelimb regeneration, we performed *t* tests to identify Affymetrix probesets (probes) that yielded significantly different gene expression estimates as a function of time. We compared average expression of each probe at day 0 to corresponding estimates obtained at each of 19 post‐amputation time points (Fig. 4). We plotted statistically significant test results against time to identify general features of the transcription program. Overall, we identified 7663 probes that yielded significantly different expression estimates between day 0 and any other time point. The number of significant probes increased with time, although the distributions differed in the direction of change from day 0; in other words, there were differences between probes that registered increasing (“up probes”) versus decreasing (“down probes”) transcript abundances. The number of up probes increased from *N* = 506 at day 0.5 to *N* = 2318 at day 24. In comparison, the number of down probes increased from *N* = 163 at day 0.5 to *N* = 1701 by day 3, and then remained relatively constant throughout the experiment. Overall, these results show that the number of significant expression changes increased with time and especially so for up probes. Assuming transcriptional patterns eventually return to baseline levels as tissues attain pre‐amputation structure and function, our sampling program clearly did not approach the terminal stages of regeneration.

**Figure 5:**
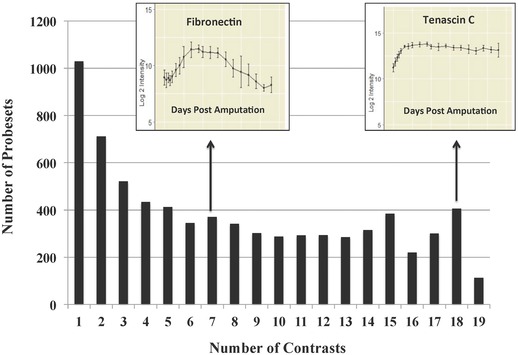
The number of times that each of the significant probesets was identified as significant when contrasting post‐amputation samples with the day 0 sample. For example, the probeset that corresponds to fibronectin yielded seven significant results out of a possible 19 contrasts (day 0 vs. day 0.5, day 0 vs. day 1, …, day 0 vs. day 28). In comparison, the probeset of tenascin C showed a significant deviation from day 0 early in the time series and thus yielded 18 significant results.

While Figure 4 shows that limb regeneration is associated with an increasing number of gene expression changes over time, it does not provide information about the number of probes that contribute to the overall distribution. Consider that the number of significant test results in Figure 4 (*N* = 59,476) is much greater than the total number of probes identified as significant (*N* = 7663). This indicates that some number of probes yielded more than one significant result across the time series. To investigate this further, we determined the number of times each probe was identified as significant across all time points (Fig. 5). Approximately 14% of the probes were only differently expressed at a single time point, and, of these, 4.6% were differently expressed at the first time point. Thus, 6331 probes were differently expressed at multiple time points. Figure 3 shows temporal profiles for 12 probes that were identified as significant for a range of different time points. These overlays show a diversity of profiles and provide an initial glance at the complexity of the overall data set. For many patterns, statistical significance is maintained across contiguous time points, although some patterns are clearly episodic.

**Figure 6:**
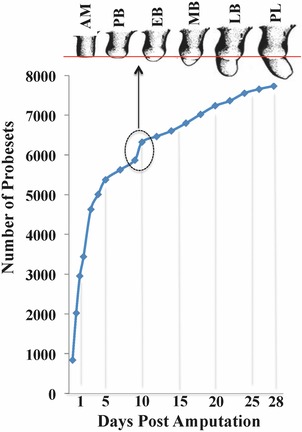
Cumulative frequency distribution showing when each of the probesets identified as significantly differently expressed when contrasting post‐amputation time points to day 0 were first identified as significant. The dashed circle shows a discontinuity in the profile between days 9 and 10 which corresponds to the onset of the early bud (EB) stage of regeneration.

A cumulative frequency plot was compiled to show the earliest time that each of the 7663 significant probesets were differently expressed in the time series (Fig. 6). The number of significant probes discovered during the first 5 days of limb regeneration increased precipitously. By 5 DPA, >5000 different probes were identified as differently expressed. Because these probes were designed to primarily target different genes, this plot clearly shows that the initial stages are transcriptionally complex from a gene expression perspective. Between days 5 and 9, the slope of the plot decreased as fewer unique probes were expressed differently per unit time. However, an upward inflection in the plot was observed between 9 and 10 DPA, indicating a qualitatively distinct increase in differential gene expression (*N* = 354 up probes; *N* = 84 down probes) for the transition between PB and EB (Fig. 6). We note that many (*N* = 97) of the up probes exhibited more than a two‐fold change in expression and showed similar patterns of expression across subsequent samples (see below). After 10 DPA, the same relative number of new probes was identified among samples. Overall, the cumulative frequency plot shows that most of the probes identified as differently expressed in the study deviated within the first 5 days of limb amputation. After this time, the number of new differently expressed genes increased more linearly over time, excepting the uptick observed between 9 and 10 DPA.

**Figure 7:**
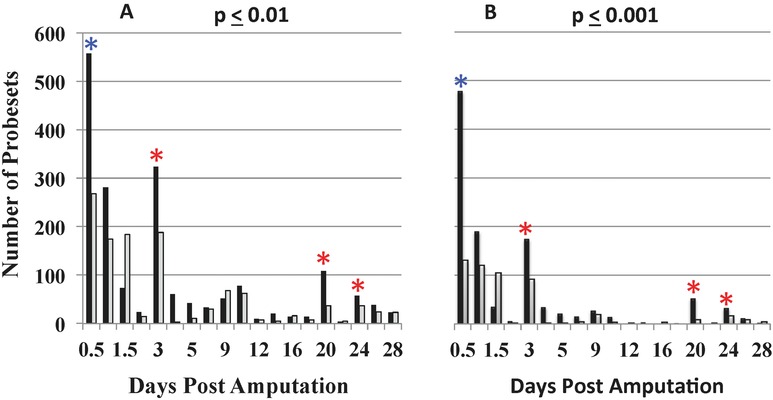
(A) Number of significant probesets identified between time adjacent samples using a *P* value threshold of 0.01 to evaluate the significance of independent *t* tests. Bars show probesets with increasing (black bars) and decreasing (grey bars) changes in abundance relative to the previous sample. The red asterisks show where the number of significant probesets increased significantly between time adjacent contrasts. For example, the number of significant probesets identified for the 2−3 DPA contrast was significantly higher than the number identified for the 1.5−2 DPA contrast. The blue asterisk at day 0.5 highlights the large number of significant probesets that were identified between the non‐amputated state and the first post‐amputation sample. (B) Number of significant probesets identified between time adjacent samples using a *P* value threshold of 0.001 to evaluate the significance of independent *t* tests. Note that punctuated episodes of transcription are observed at a more conservative statistical threshold. All other components of the figure follow the descriptions above.

To further examine temporal changes in transcription, we performed *t* tests between all adjacent time points (e.g., day 0 vs. day 0.5, day 0.5 vs. day 1, …, day 26 vs. day 28) (Fig. 7). In contrast to the approach above, where day 0 values were compared to all subsequent time points, these comparisons identified time intervals where the total number of significant probes increased or decreased as the limb regeneration program unfolded. The temporal distribution of significant probes from this analysis was different from the pattern described above (compare with Fig. 4). The greatest number of significant probes was identified for the first contrast between non‐amputated limb and 0.5 DPA (day 0−0.5, *N* = 669). After this time, the number of significant probes declined and increased over short intervals of time, with the overall pattern revealing four peaks of transcription across the time series: 0−1 DPA, 2−3 DPA, 18−20 DPA, and 22−24 DPA. We note that these peaks are associated with statistically significant increases in the number of significant probes identified between time adjacent contrasts (McNemar's test was evaluated at a Bonferroni adjusted threshold *P* < 0.0028). For example, the number of significant probes identified for the day 2−3 contrast is approximately 10 times higher than the day 3−4 contrast. More up than down probes were identified for each peak and fewer significant probes were associated with the later three peaks. The 0−1 DPA peak associates with limb amputation, the 18−20 DPA peak with the onset of the LB stage, and the 22−24 DPA peak with the pallet stage. This distribution suggests that the process of limb regeneration is punctuated by transcriptional changes that correlate with morphological and histological changes described by Tank et al. (1976).

### Similarity of microarray samples

**Figure 8:**
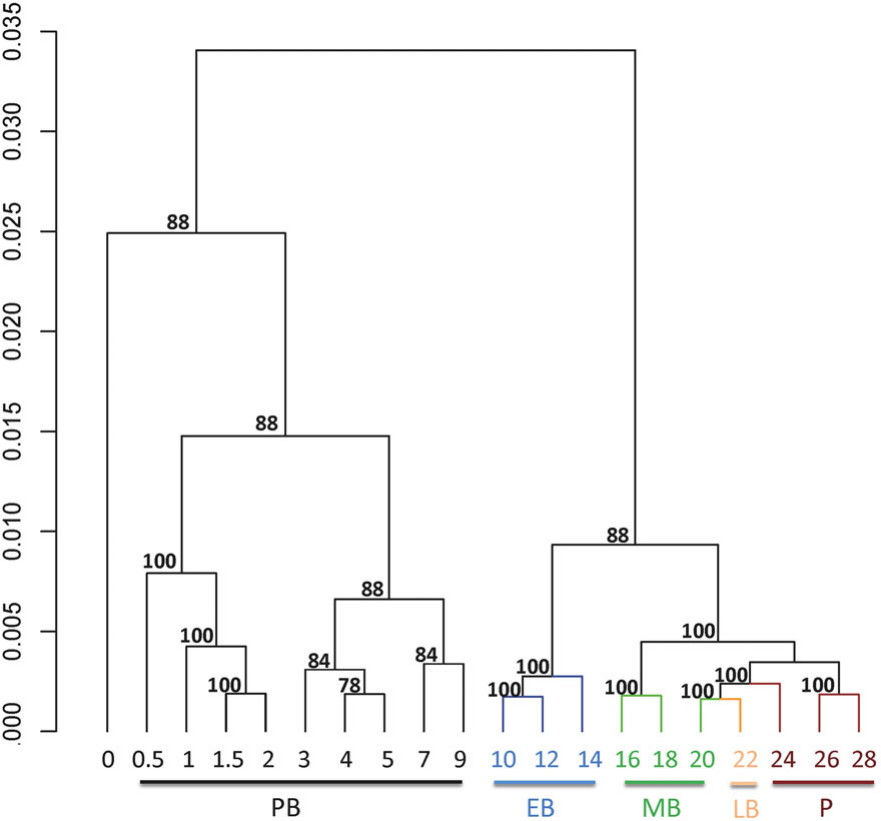
Hierarchical cluster analysis reveals temporal grouping of samples, with the first bifurcation splitting samples into early (days 0−9) and late (days 10−28) groups. The numbers positioned at nodes in the dendogram are approximately unbiased bootstrap values. The *y*‐axis is 1 − Pearson's correlation coefficient and the *x*‐axis shows the samples according to the day of collection. For example, 0, day 0; and 0.5, day 0.5. The colors refer to regeneration stages: pre‐bud (PB), early bud (EB), medium bud (MB), late bud (LB), and pallet (P).

We used hierarchical clustering to determine which time points in the experiment were more similar (Fig. 8). In general, we found that time adjacent samples clustered more closely together. This result supports the idea that overall gene expression diverges gradually over time during limb regeneration. We expected the first major split to partition the non‐amputated day 0 sample from all other samples, because the day 0 sample presents the most dramatic morphological contrast. Unexpectedly, the first or highest bifurcation in the dendrogram split the microarray samples into two groups: a day 0−9 group and a day 10−28 group. This result was also obtained when hierarchical clustering was performed with a jackknifing procedure (Fig. S1). Thus, in addition to the peaks of transcription described above, transcriptional differences between 9 and 10 DPA are sufficiently different to implicate this as a critical transition point in the limb regeneration program.

**Table 1:** List of significantly enriched gene ontology (GO) terms that were identified for five post‐amputation time intervals (DPA, days post‐amputation)

GO Term	# Genes	Prob
0–1 DPA		
GO:0030574∼collagen catabolic process	8	0.002
GO:0006928∼cell motion	39	0.002
GO:0009719∼response to endogenous stimulus	35	0.002
GO:0009991∼response to extracellular stimulus	23	0.008
GO:0042127∼regulation of cell proliferation	46	0.037
GO:0006916∼anti‐apoptosis	21	0.016
GO:0051252∼regulation of RNA metabolic process	78	0.025
GO:0051094∼positive regulation of developmental process	22	0.043
GO:0031328∼positive regulation of cellular biosynthetic process	40	0.043
GO:0031667∼response to nutrient levels	20	0.027
GO:0001944∼vasculature development	22	0.027
GO:0042330∼taxis	12	0.037
GO:0006952∼defense response	30	0.038
2–3 DPA		
GO:0007049∼cell cycle	56	6E‐07
GO:0000279∼M phase	27	6E‐04
GO:0051276∼chromosome organization	31	2E‐03
GO:0006259∼DNA metabolic process	37	1E‐04
9–10 DPA		
GO:0031012∼extracellular matrix	15	6E‐06
GO:0005576∼extracellular region	25	2E‐04
GO:0005604∼basement membrane	7	5E‐04
18–20 DPA		
GO:0051789∼response to protein stimulus	9	0.002
22–24 DPA		
GO:0008202∼steroid metabolic process	10	8E‐07

### Identification of genes and associated biological processes for significant temporal intervals

Overrepresentation analyses were performed to identify significantly enriched biological process gene ontology (GO) terms for the day 9−10 interval and the four intervals where the number of differently expressed probes increased significantly (day 0−0.5; days 2−3; days 18−20; days 22−24). Given the small number of significant genes identified for some intervals, gene lists were also curated manually through literature searches. Additionally, to facilitate the discovery of groups of genes with associated patterns of expression, Pearson's correlation coefficient was calculated between genes using transcript abundance estimates across the entire time series.

#### The 0−1 DPA interval

**Figure 9:**
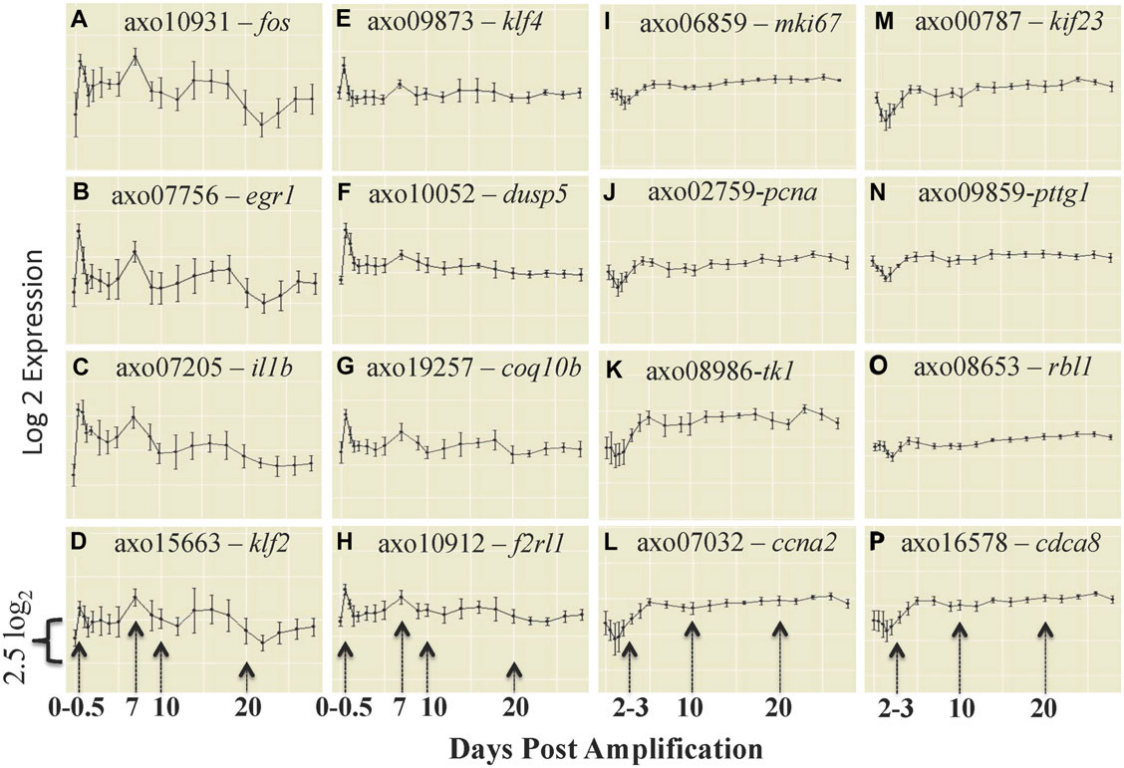
Expression profiles for genes identified as differently expressed at 0−0.5 DPA (A−H) and 2−3 DPA (I−P). Each estimate of transcript abundance was calculated from 10 replicate samples. The error bars are standard deviations.

The presumptive axolotl−human gene orthologs (*N* = 841) identified as differently expressed at the first post‐amputation time point significantly enriched several GO terms, including cell motion, response to endogenous and extracellular stimulus, collagen catabolism, defense, RNA metabolism, anti‐apoptosis, and regulation of development and cellular biosynthetic processes (Tables 1 and S1). The genes that enriched these terms suggest that limb amputation induces transcriptional changes beyond a simple wound healing response. Transcript levels for >70 genes encoding transcription factors and components of developmental signaling pathways were expressed differently after amputation, and these genes showed increasing (e.g., *areg*, *bmp2*, *cyr61*, *egr1*, *fos*, *id1−2*, *klf2−4*, *klf10*, *myc*, *nr4a1−3*, *sall4*, *spi1*) and decreasing changes in transcript abundance (e.g., *hoxc8*, *lmx1b*, *mycn*, *nr2f2*, *prickle1*). Many of the presumptively upregulated genes at 0.5 DPA, including immediate early genes (e.g., *fos*, *egr1*, *il1b*, *cry61*), exhibited a unique and significant increase in transcript abundance at day 7. Strikingly, these immediate early genes exhibited similar expression profiles across the entire 28‐day time series, a pattern that is suggestive of transcriptional co‐regulation and temporal re‐deployment of gene regulatory networks during regeneration (Fig. 9A−H).

#### The 2−3 DPA interval

A second, punctuated phase of gene expression was detected between 2 and 3 DPA. The 435 annotated genes that were identified for this interval enrich terms associated with mitosis, DNA replication, and more generally the cell cycle (Tables 1 and S1). This set included cyclins (*ccna2*, *ccne2*, *ccng2*), regulators of DNA replication (*cdc6*, *cdc7*), nucleotide synthesis (*rrm1*, *rrm2*), DNA polymerization (*pola2*, *pole2*), chromosome condensation (*smc2*, *smc4*), and chromatin organization (*smarcad1*, *smarce1*, *smarca5*, *smarca2*). The majority of these genes, including markers of cell proliferation (*pcna*, *mki67*, *tk1*), increased during this interval, and after this time transcript abundances increased gradually or remained relatively constant throughout the 28‐day time course (Fig. 9I−P). The increase in transcript abundance for many genes followed a pattern of decreasing transcript abundance between 0 and 2 DPA. Thus, 2−3 DPA marked a transition in the limb regeneration program, with a punctuated increase in cell cycle associated transcripts indicating a change in the number of proliferative cells within the distal limb stump.

#### The 9−10 DPA interval

**Figure 10:**
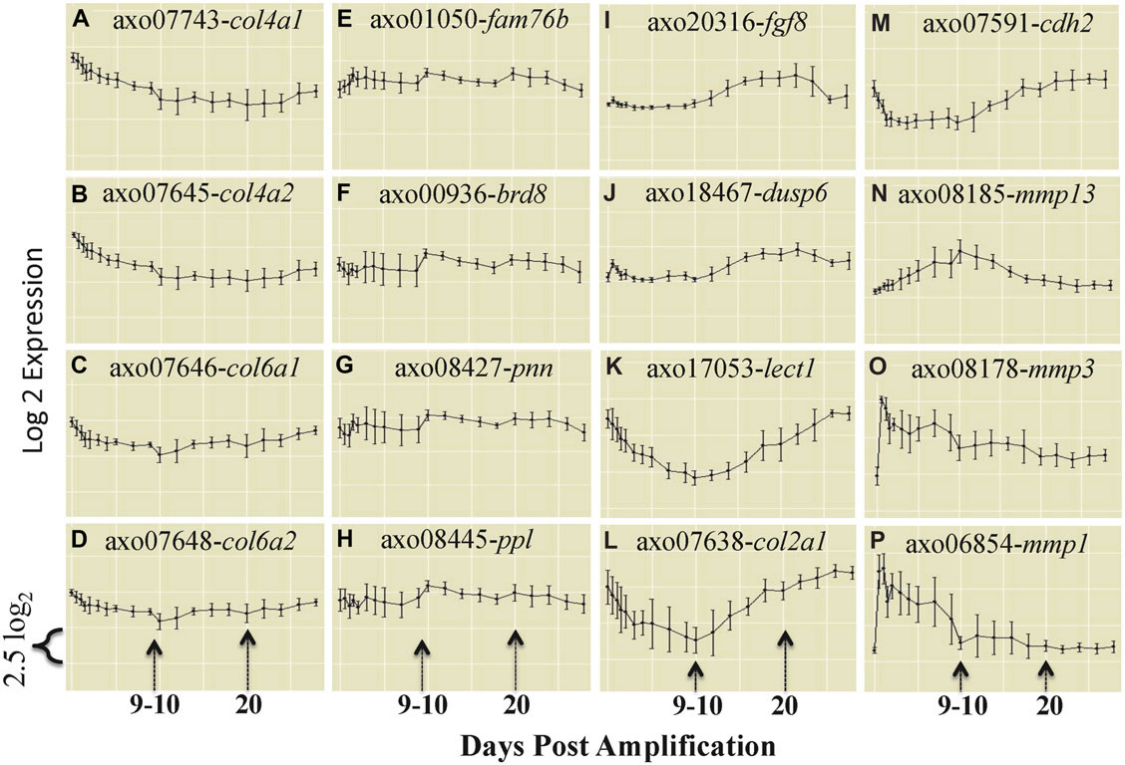
Expression profiles for genes identified as differently expressed at 9−10 DPA (A−H). Additional genes that are associated with FGF signaling (I, J), chondrogenesis (K, L), and osteoclast activity (M, N) showed expression profile transitions at 9−10 DPA. Each estimate of transcript abundance was calculated from 10 replicate samples. The error bars are standard deviations.

Hierarchical clustering of microarray samples identified the 9−10 DPA interval as the most distinct in the overall limb regeneration transcriptional program (Fig. 8). In part, because the list of differentially expressed genes with annotations was small and functionally diverse for this interval (*N* = 117), no significantly overrepresented biological process terms were identified. Manual inspection of these genes predict that many encode extracellular matrix and basement membrane proteins; indeed extracellular matrix, extracellular region, and basement membrane were identified as significantly enriched cellular component GO terms (Table 1). The majority of these genes showed a decrease in transcript abundance for this interval, with 10 DPA estimates representing the lowest calculated mean in the overall 28‐day expression profile. And these genes showed highly similar expression profiles (Fig. 10A‐D). Using hierarchical clustering, additional genes with correlated expression profiles were identified, including groups that showed an increase in transcript abundance for the 9−10 DPA interval. These genes were associated with several different functions, including chromatin remodeling and transcriptional regulation (*brd8*, *ep300*, *nipbl*, *phf6*, *rarg*, *smarcc1*, *smc4*, *zmynd11*), vesicle formation and intracellular/intercellular trafficking (*golga4*, *gcc2*, *clip1*, *cltc*, *itsn2*, *kif21a*, *plekhf2*), tight junctions/desmosomes (*dsp pnn*, *ppl*), cell proliferation and growth control (*efna1*, *ell3fap1*, *fap*, *ppp2r5c*, *samd9l*, *scaf1*, *tet2*, *tp53inp1*, *znf217*), and neural‐associated functions (*avil*, *epb41l1*, *map1b*, *plxnb2*, *slitrk6*, *znf592*). In addition to conservation of mean expression estimates for genes across the 28‐day time course, note that the variance in expression changed coordinately before and after 9−10 DPA (Fig. 10E−H).

**Figure 11:**
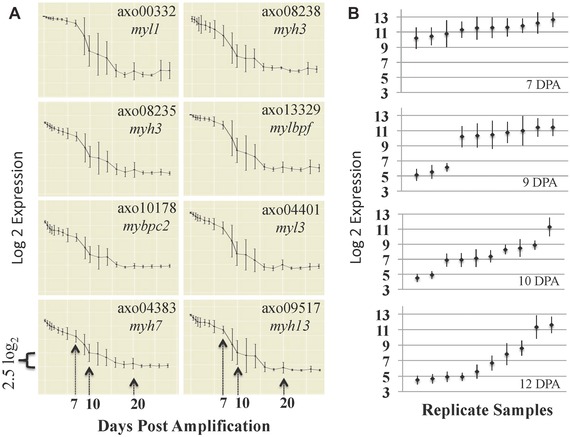
(A) Expression profiles of muscle‐associated genes showing correlated changes in average transcript abundance and standard deviation. (B) The expression estimates for the eight muscle genes in (A) were used to calculate an among genes average for each tissue sample that was collected at 7, 9, 10, and 12 DPA. The replicates are ordered within figures to show the distribution of estimates from low to high. The error bars are standard deviations.

We suspected that other genes showed transitions in expression at 9−10 DPA, but the magnitude of change was not sufficiently large within this temporal window to eclipse statistical and fold change thresholds. To identify these genes, a web‐accessible tool was developed to manually view expression profiles on Sal‐Site (http://www.ambystoma.org). We identified several groups of genes with expression patterns that transitioned approximately at 9−10 DPA, including genes associated with fibroblast growth factor (FGF) signaling (*fgf8*, *dusp6*, *spry1*, *flrt3*), osteoclast activity (*acp5*, *calb1*, *ctsk*, *mmp13*, *ostm1*, *tcirg*), and chondrogenesis (*cdh2*, *chodl*, *cilp*, *col2a1*, *col9a1−3*, *lect1*, *col2a1*, *ogn*) (Fig. 10I−P). Also, genes associated with muscle cell structure and function showed correlated decreasing patterns of transcript abundance and highly variable estimates of mean expression from 9 to 12 DPA (Fig. 11A−H). Further inspection of these muscle genes revealed a complex pattern of variation. Estimates of transcript abundance were highly correlated within tissue replicates at 7−12 DPA, but estimates varied among replicates (Fig. 11B). In other words, the decline in transcript abundance varied among tissue samples collected on 9, 10, and 12 DPA. Overall, these and the results above show that the 9−10 DPA interval marked a transition point in regeneration, where transcript levels coordinately changed within and among a variety of tissues and cell types.

#### The 18−20 DPA interval

**Figure 12:**
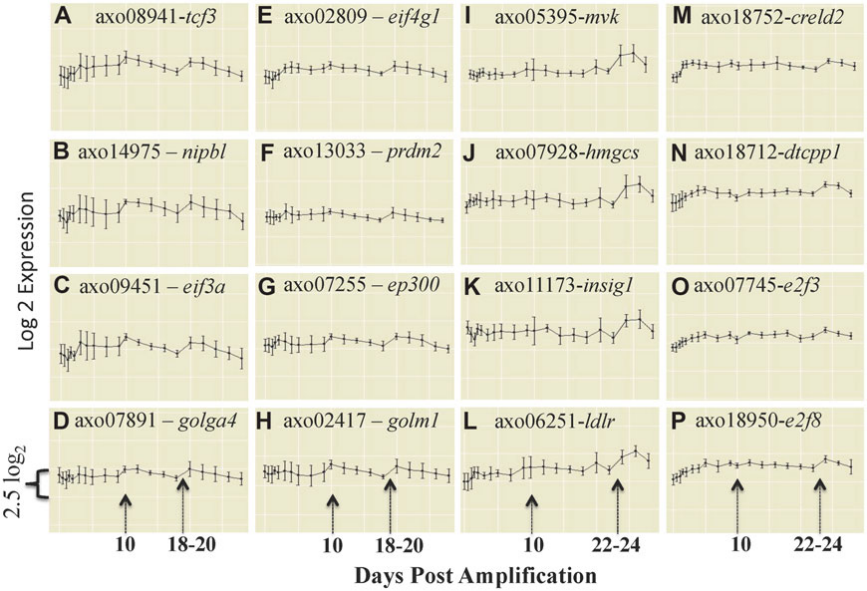
Expression profiles for genes identified as differently expressed at 18−20 DPA (A−H) and 22−24 DPA (I−P). Each estimate of transcript abundance was calculated from 10 replicate samples. The error bars are standard deviations.

The 150 annotated genes that were identified for this interval only significantly enriched a single GO term—response to protein stimulus. Three of these were immediate early response genes that exhibited the same temporal expression profile across the 28‐day time series (Fig. 9A−C). As was identified for the 9−10 DPA interval, several genes are predicted to function in chromatin dynamics, chromatin remodeling, and transcriptional regulation (*ahctf1*, a*trx*, *dnmt1*, *ep300*, *nipbl*, *rreb*, *sass6*, *setd2*, *tcf3*, *smc4*, *tlk1*, *vezf1*, *zmynd11*, *znf638*, *znf644*), vesicle formation and intracellular/intercellular trafficking (*golga4*, *golm1*, *clip1*, *cltc*), and tight junctions/desmosomes (*ap2b1*, *dsg2*, *dsp*, *pvr2*). Additionally, genes associated with transcriptional elongation (*aff4*, *eif3a*, *eif4g1*) and retinoblastoma‐mediated tumor suppression (*rbbp8*, *prdm2*) exhibited a significant increase in expression at 20 DPA. When we compared the expression patterns of these genes, we were surprised to find that almost all presented the same change in average expression and standard deviation across the 28‐day time course. For example, *tcf3*, *eif4g1*, *ep300*, *nipbl*, *golga4*, *golm1*, *eif3a*, and *prdm2* exhibited flat and variable expression estimates among samples from days 3 to 9, expression increased between days 9 and 10, and then after this time through day 20 average expression decreased and estimates were more precise (Fig. 12A−H). These patterns suggest that many of the 18−20 DPA genes are coordinately regulated.

#### The 22−24 DPA interval

The annotated genes (*N* = 80) identified for this interval enriched cholesterol and steroid metabolic GO terms (Table 1). All of these genes showed increasing transcript abundances (*cyp51a1*, *fdps*, *hmgcs1*, *ldlr*, *lss*, *mvk*, *pmvk*, *insig1*, *sc4mol*, *sc5dl*, *sqle*) (Fig. 12I−L). Additionally, several additional genes associated with phospholipids, lipoproteins, and fatty acids showed increasing transcript abundances (*aacs*, *acsl4*, *etnk1*, *isyna1*, *lipg*), as well as genes with functions associated with the endoplasmic reticulum (*dnajc3*, *dnajb9*, *hyou1*, *kdelr3*, *sdf2l1*), the site of lipid and steroid biosynthesis. Although not identified as significantly overrepresented GO terms, genes associated with the cell cycle (*cdkn1b*, *e2f3*, *e2f8*, *frk*, *nupr1*, *thap5*), osteogenic differentiation (*creld2*), and deoxyribonucleotides (*dctpp1*, *dck*, *tk1*) were also expressed differently (Fig. 12M−P). Overall, the genes identified at 22−24 DPA were biased for steroid metabolism, and as was detailed for genes identified for the previous intervals of punctuated gene expression, genes were coordinately regulated throughout limb regeneration.

## Discussion

We described results from an experiment that was designed to comprehensively profile gene expression during the first 28 days of axolotl limb regeneration. We reduced the overall complexity of the data set by drawing focus to specific intervals of time where gene expression changed most dramatically. As detailed above, the intervals that we identified show correspondence to biological processes and developmental stage transitions in the regeneration series presented by Tank et al. (1976). By linking transcription with well‐described morphological and histological events of regeneration, we have built an integrative framework to generate hypotheses and guide subsequent phases of data analysis. Below, we discuss transcriptional insights and then integrate into this framework the morphological and histological events described by Tank et al. (1976).

### Salamander limb regeneration is transcriptionally complex

Microarray experiments typically identify hundreds to a few thousand differently expressed genes when using three to five replicate arrays. With the extra statistical power that comes with using 10 replicate arrays per time point, we identified over 7000 differently expressed genes when comparing post‐amputation samples to the non‐amputated day 0 sample. The largest number of significant probes was identified for the first post‐amputation time point, and then the overall number of probes steadily increased with time. This pattern is explained by the following: (1) some genes that were expressed differently immediately after limb amputation continued to be differently expressed across subsequent time points; (2) some genes that were not expressed differently early were expressed differently at later post‐amputation time points (see Fig. 3 for examples). As a result of these two patterns, the regeneration transcriptional program diverges progressively from the non‐amputated state over time. Because of progressive divergence, it is not possible to identify transitions in the regeneration program using only contrasts to the non‐amputated, day 0 sample. This is a simple but important point to consider when conducting statistical tests to identify differently expressed genes. Contrasting post‐amputation expression values to day 0 does not provide insight about transcriptional change over time. Thus, we also performed statistical contrasts between all adjacent time points. The results from these contrasts revealed the pattern of transcriptional change to be mostly gradual over time, with punctuated episodes of transcription at specific time intervals.

### Importance of fine temporal sampling

Knapp et al. (2013) recently proposed that transcription during axolotl limb regeneration is organized into three distinct temporal phases—early (0−12 h), middle (24−168 h), and late (288−528 h). These phases were identified using hierarchical clustering of microarrays, performed in our study to group samples according to overall gene expression similarity. If we arbitrarily slice our dendrogram just below the third bifurcation from the top (Fig. 8), we generate subgroupings that support the temporal phases identified by Knapp et al. (2013). Such agreement is expected given the results discussed above—with the overall gene expression program diverging over time and with gradual divergence of time adjacent samples, temporal structuring of the data will be evident regardless of the number of samples collected. However, our study shows that, with finer sampling, more temporal structuring is revealed, including the bifurcation that split PB samples from samples collected after day 9. While statistical groupings may not have biological relevance, our results show that sparse and shallow sampling will always underestimate the overall complexity of the limb regeneration program and miss critical intervals of transcriptional change.

### Episodes of transcriptional activity correlate with stages of regeneration

By contrasting all time adjacent microarray estimates (Fig. 7), we identified four intervals of time where the number of differently expressed probes reached local maxima in the time series. The first and highest of these peaks in transcription occurred at 0.5 DPA, the earliest sample in the data set. Approximately twice as many of the probes identified at this time showed an increase in transcript abundance (rather than a decrease) relative to the day 0 sample. This pattern has been observed in other gene expression studies of tissue regeneration and wound healing, and has been attributed to changes in transcriptional regulation within surviving cells of the limb stump and changes in the abundance of cells that actively migrate to the injury site (Monaghan et al. 2009, 2012; Knapp et al. 2013; Stewart et al. 2013). Following this peak, the number of probes identified as differentially expressed decreased significantly, with only 14 significant probes detected between 1.5 and 2 DPA. Then, in the next interval of time (2−3 DPA), a second episode of transcriptional change occurred, again with the majority of probes measuring increasing rather than decreasing transcript abundances. The increase of significant genes detected between 2 and 3 DPA suggests there is a precise signaling event that orchestrates a broad and coordinated transcriptional output. Tank et al.'s (1976) description of the histology after the initial wound healing response does not implicate specific biological processes that would be consistent with a broad, activated, transcriptional response. Our GO analysis found that many of the genes that were upregulated for this interval are predicted to encode proteins that have cell cycle functions, which is consistent with the findings of Maden (1978) concerning the onset of cell proliferation during limb regeneration and the microarray study of Knapp et al. (2013). It is interesting that cell cycle transcript levels only showed a punctuated increase very early after limb amputation and not at a time that more closely preceded limb bud outgrowth and blastema formation. Cell cycle gene expression remained relatively constant after 5 DPA with some transcript levels approximating pre‐amputation levels and others showing gradual increases during limb bud outgrowth. It seems likely that the increase in cell cycle transcripts at 2−3 DPA reflects an increase in mitotic activity across multiple cell types, including immunological and osteoclast progenitors, epithelial cells within and bounding the margins of the wound epidermis, and blastema progenitors.

Subsequent episodes of transcription were not as prominent as the 0−0.5 and 2−3 DPA peaks, but they did coincide with the onset of regeneration stages defined by Tank et al. (1976). The peak identified for the 18−20 DPA peak coincided with the transition from MB to LB stage. The gross morphological changes associated with LB are mostly quantitative, a continuation of bud outgrowth that initiated at 10 DPA. The bud becomes flattened and asymmetrical in shape and blood vessels are present. At a histological level, Tank et al. (1976) found very few osteoclasts relative to earlier stages, cartilage was being synthesized adjacent to bone, and cartilage and muscle progenitor cells were organized as condensations. Few new significant transcripts were identified between 20 and 22 DPA when the majority of individuals were classified as LB. Then, coinciding with onset of the morphologically distinct pallet stage, a final transcriptional peak was observed. During this stage, the digit primordial sequentially becomes visible and, histologically, cartilage and myoblasts are beginning to differentiate. Because we collected tissue from the distal tip of the elongated palette, transcripts from proximal muscle tissue and muscle progenitor cells were not collected. The 20−22 DPA interval was significantly enriched for gene expression changes that associate with steroid/cholesterol metabolism. Sterols and lipids are required for development of bones in chicken appendages during embryogenesis (Schmidt et al. 2009). Several of the genes (*fdft1*, *fdps*, *hmgcs1*, *insig1*, *ldlr*, *lss*, *mvk*, *sc4mol*, *sc5dl*, *sqle*) that Schmidt et al. (2009) identified as differently expressed in response to conditional knockout of *por* in mice, a cytochrome P450 that is essential for steroidogenesis (Flück et al. 2004), were coordinately upregulated in our study at 22−24 DPA. If these genes are similarly upregulated during patterning of the axolotl limb, it would provide a striking example of developmental recapitulation at a gene regulatory level.

Although not identified as a significant peak of transcriptional activity, the 9−10 DPA interval is an important integration point in the limb regeneration program based on several lines of evidence. First, microarray samples before and after this interval were resolved into different groupings at the highest level of our hierarchical cluster dendrogram (Fig. 8). Second, a relatively higher number of probes registered a significant difference in transcription for this interval than flanking intervals (Fig. 6). Finally, had we tested for significant decreases in the number of transcripts between time adjacent contrasts (Fig. 7), the 10−12 DPA interval would have been identified as significant. In our study, the 9−10 DPA interval marked the transition of individuals from the PB stage to the EB stage. Tank et al. (1976) observed that the EB transition is associated with maturation of the wound epidermis and especially the basal cell layer, which becomes well organized and presumably functional to release signaling factors to blastema cells. Blastema cells accumulate and increase in number, and the amputated stump shows the first visible signs of outgrowth. Also, this transition marks a peak in the activity of osteoclasts adjacent to bone. We did identify genes associated with chromatin remodeling, transcriptional regulation, cell adhesion, vesicles and cellular trafficking, FGF signaling, and chondrogenesis that increased in abundance at this time. Also, we observed a decrease in transcription of genes associated with extracellular matrix remodeling and differentiated muscle cell phenotypes. The coordination of these events implicates the PB−EB transition as a nexus of limb regeneration.

### Correlated changes in transcript abundance during limb regeneration

We found that genes associated with specific, punctuated episodes of transcription tended to show similar expression profiles across the entire 28‐day time course of limb regeneration. There are at least three explanations for observing groups of genes that show correlated changes in transcript abundance: (1) the genes are integral to the transcriptome of a specific cell type; (2) the genes are integral to a specific biological process that is regulated within or across cell types; or (3) the genes are integral to multiple biological processes that are coordinately regulated across different cell types. Consistent with the first explanation, we observed a decline in transcript abundance during the PB stage for genes that code for muscle structural and functional proteins. This pattern of decline has been observed in other studies (Monaghan et al., 2009; Knapp et al. 2013); however, because our experimental design deeply sampled post‐amputation time points, we discovered something novel—estimates of transcript abundance for muscle‐ associated genes were highly correlated within tissue sample replicates, with replicates yielding high or low estimates across genes. This pattern of variation suggests that muscle transcripts decrease variably among samples as fibers presumably undergo histolysis in the limb stump. The biological significance of this finding is unclear at this point, but coordinate regulation in this case may trace to a homeodomain protein (*six1*) that was recently shown to regulate fast muscle gene expression via coordinate regulation of a long noncoding RNA (Sakakibara et al. 2014). It is important to note that this unusual pattern of gene co‐regulation was detected because time points were deeply sampled in our study.

Patterns of gene co‐expression may also reflect the activation of gene sets that collectively orchestrate specific biological processes, within or across cell types. It is known for example that FGF signaling during limb bud outgrowth encompasses basal cells of the wound epidermis and underlying blastema cells (Han et al. 2001). Clearly, it will be important to determine the cellular location of transcripts from this and other studies and integrate all of this information within the temporal limb transcriptional model established here. Towards these goals, the transcriptional data provide context for generating testable hypotheses that are most likely to enrich the model and thus understanding of limb regeneration. For example, determining the cellular location of 22−24 DPA transcripts would potentially identify differentiating chondrocytes and establish a new animal model for investigating steroid/cholesterol metabolic genes in bone development and patterning. Additionally, determining the cellular location of 9−10 DPA transcripts, as a first step, would provide insight about cues that integrate multiple biological processes within narrow windows of developmental time. The transcriptional model presented here and hypotheses that arise from this model provide an integrative framework for future studies of limb regeneration.

## Materials and Methods

### Animal procedures

A total of 200 *A. mexicanum* were obtained from the *Ambystoma* Genetics Stock Center at the University of Kentucky. Animals were maintained at 17−18°C and fed California blackworms once per week. Rearing water (40% modified Holtfreter's solution) was changed three times per week. Animals were reared to between 5 and 8 cm snout−vent length for tissue collection. The ages of animals at surgery were between 6 and 10 months. Axolotls were anesthetized in 0.01% benzocaine and sterile razor blades were used to perform amputations at mid‐zeugopod and mid‐stylopod positions. Limb bones were not trimmed back after performing amputations. A single investigator performed all the amputations. The handling and surgical manipulation of axolotls was carried out according to University of Kentucky Animal Care and Use guidelines.

### RNA extraction and microarray analysis

Exactly 1 mm of the distal‐most tissue was collected from amputated limbs at 20 different time points during limb regeneration. Ten tissue samples were collected from amputations of the stylopod, and 10 from zeugopod amputations at each time point. RNA was extracted for individual samples using Trizol Reagent (Invitrogen, Carlsbad, CA) followed by RNeasy mini kit (Qiagen, Valencia, CA). RNA quality was assessed by spectrophotometry using Nanodrop ND‐1000 (Nanodrop, Wilmington, DE) and run on a Bioanalyzer 2100 (Agilent, Santa Clara, CA). The same investigator who performed amputations collected all the tissues and isolated RNA. Microarray hybridization using an *Ambystoma* Affymetrix array (Huggins et al. 2012) was performed by the University of Kentucky Microarray Core Facility. The raw microarray data (.CEL files) and the microarray annotations are available at Sal‐Site (http://www.ambystoma.org/genomeresources).

### Statistics

Several quality control methods were used to examine expression values across the 200 GeneChips in the experiment. Box plots were generated in Expression Console (Affymetrix, Santa Clara, CA) to examine the consistency of expression across arrays, and principal components analysis and Mahalanobis distances were calculated in JMP to examine array clustering in multivariate space. None of the arrays appeared to be outliers so all were retained for normalization using Affymetrix Expression Console software to accomplish the robust multichip averaging (RMA) algorithm (Irizarry et al. 2003).

Two tailed *t* tests assuming heteroscedasticity were performed on log_2_ transformed signal intensities. Two sets of contrasts were performed. The first set compared the mean for each post‐amputation time point to the day 0 sample mean. The second set compared sample means between each adjacent time point. Correcting for multiple testing left several choices. The traditional approach is to calculate a false discovery rate (FDR) Benjamini and Hochberg, 1995 for the full statistical model, which in this case tests each probeset for a significant difference as a function of sampling time. Alternatively, FDR could be calculated post hoc after performing all *t* tests (19 contrasts × ∼20,000 probesets = ∼380,000 tests). We reasoned that the traditional approach would be over‐powered for our experimental design with 199 degrees of freedom and indeed this approach identifies over half of the probesets as statistically significant. Conversely, correcting for ∼380,000 tests is overly conservative and probably removes many true positives. We consequently chose to control the probability that a significant probeset is a false positive at *P* < = 0.01 for each *t* test. A fold change cutoff of 1.5 (log_2_ difference between means of 0.58) was utilized within *t* tests to remove low magnitude expression changes.

For comparisons between adjacent time points, a cutoff of *P* = 0.001 was also evaluated (Fig. 7B) as this *P* value was found to be closer to the common cutoff of an FDR of α = 0.05 for several of the comparisons. Many of the comparisons relative to the initial time point pass an FDR of α = 0.05, and therefore a cutoff of *P* < 0.001 was not considered. A fixed *P* value was selected rather than an FDR value because the FDR becomes more stringent as fewer true positives are present in the data set, given that the FDR calculates the proportion of expected false positives rather than the probability of a given gene being a false positive. The FDR correction does not take into account that a gene near the statistical cutoff has a higher probability of being a false positive than a gene that has a *P* value near zero. Not all time points were expected to show the same number of changes relative to the previous time point or the initial time point. Therefore, we chose to fix the *P* value cutoff and report the expected false positive rate, rather than fix the FDR at the level of each comparison.

We statistically compared the number of genes identified from time adjacent contrasts to identify intervals of time where transcription showed punctuated patterns of change. For example, the numbers of significant probesets identified for the 0−0.5 and 0.5−1 DPA contrasts were compared to test for a significant increase in the number of significant probesets identified for the later time contrast. We used McNemar's test in R (McNemar 1947; R Core Development Team 2008) to perform this analysis and evaluated significance at a Bonferroni adjusted *P* value threshold of 0.0028 because 18 statistical tests were performed (α = 0.05/18 = 0.0028).

For each probeset on the Affymetrix Amby002 microarray, average expression estimates and standard deviations were plotted as a function of time, and the resulting profiles were compiled into a searchable database at Sal‐Site (www.ambystoma.org). The profiles are searchable by gene name, official gene symbol, or Affymetrix probeset ID. Profiles were made using the ggplot2 (Wickham 2009) package in R (R Core Development Team 2008).

Cluster analysis and bootstrapping were performed using the pvclust package (Suzuki and Shimodaira 2006). For each probeset, average log_2_ signal intensity was calculated for each time point, and these data were analyzed using the pvclust function which performs hierarchical clustering and approximately unbiased bootstrapping. Default parameters were used, including average linkage clustering on 1 – Pearson's correlation. To validate the pvclust results, hierarchical clustering using average linkage on distances of 1 − Pearson's correlation was repeated using the base R package. These data were jackknifed by removing half of the probesets and performing 1000 iterations. A consensus tree was generated by PAUP version 4.0 by loading the resulting clusters in Newick format (Swofford 2003). Dendrograms were converted to Newick notation by using the ape package in R (Paradis et al. 2004).

GO analyses were performed in DAVID (Dennis et al. 2003; Huang et al. 2009a, Huang et al., 2009b) using protein GI accession numbers from the microarray annotation file as a reference gene list. Gene lists for time adjacent contrasts identified as having a high number of significant genes based on McNemar's test were used as input. A Benjamini corrected *P* value of 0.05 was used to define significantly overrepresented ontologies.

## Supporting information

Additional Supporting Information may be found in the online version of this article at the publisher's website:

**Table S1**. Probes that were identified as significantly different by contrasting time adjacent samples for five intervals during limb regeneration.

**Table S2**. Significantly overrepresented gene ontology terms identified for intervals of time during limb regeneration.

**Figure S1**. Hierarchical cluster analysis reveals temporal grouping of samples, with the first bifurcation splitting samples into early (days 0−9) and late (days 10−28) groups. The numbers positioned at nodes in the dendogram are jackknifed probability values. The *x*‐axis shows the samples according to the day of collection. For example, 0, day 0; and 0.5, day 0.5. The colors refer to regeneration stages: pre‐bud (PB), early bud (EB), medium bud (MB), late bud (LB), and pallet (P).
