# Supplementary material for: Gene expression during the first 28 days of axolotl limb regeneration I: Experimental design and global analysis of gene expression
Source: Regeneration (Oxf). 2015 Jun 19;2(3):120–36. doi: 10.1002/reg2.37 (PMC4860271; doi:10.1002/reg2.37)
Supplement: Supplementary file 1 — Table S1. Probes that were identified as significantly different by contrasting time adjacent samples for five intervals during limb regeneration. [file REG2-2-120-s001.pdf]

Supplemental Table 1. Probes that were identified as significantly different by contrasting time adjacent samples for 5 intervals during limb regeneration.

0-1 DPA		2-3 DPA		9-10 DPA		18-20 DPA		22-24 DPA	
**Probe ID**	**Gene ID**	**Probe ID**	**Gene ID**	**Probe ID**	**Gene ID**	**Probe ID**	**Gene ID**	**Probe ID**	**Gene ID**
axo00012-r	TP53INP1	axo00234-r	CD97	axo00012-r	TP53INP1	axo00610-f	NIPBL	axo00011-f	SC5DL
axo00052-r	C1orf156	axo00255-r	CLIC6	axo00277-f	COL6A3	axo00640-f	CD109	axo00296-f	ANTXR2
axo00056-r	KIAA1737	axo00286-f	CCNE2	axo00610-f	NIPBL	axo00665-f	ATRX	axo00298-f	PSAT1
axo00060-f	CTTNBP2	axo00347-f	SELM	axo00810-f	C7orf11	axo00827-r	C19orf22	axo01256-r	OSR1
axo00075-r	HK1	axo00381-f	COL12A1	axo00936-f	BRD8	axo00939-f	AP2B1	axo01498-f	GRPEL2
axo00131-r	ZNF300	axo00383-f	COL12A1	axo01050-f	FAM76B	axo00946-f	ADCY4	axo02877-f	RNF14
axo00137-f	ZNF300	axo00388-f	ACY3	axo01634-r	SAMD9L	axo01050-f	FAM76B	axo02968-f	TSC22D3
axo00180-f	HK2	axo00389-f	ACY3	axo01635-f	SAMD9L	axo01226-f	PYCARD	axo03105-f	ZNF568
axo00255-r	CLIC6	axo00677-r	RRM1	axo01795-f	COL29A1	axo01568-f	CDCA2	axo03114-r	RBM43
axo00286-f	CCNE2	axo00699-r	RRM2	axo01910-f	SIAE	axo01959-r	TRIM39	axo03266-r	KRT12
axo00349-f	SDCBP2	axo00700-r	RRM2	axo02434-r	EPB41L1	axo02417-f	GOLM1	axo04425-f	SLC7A3
axo00407-f	TREX2	axo00701-r	RRM2	axo02525-f	PPP2R5C	axo02434-r	EPB41L1	axo04869-f	ZNF154
axo00457-r	MXI1	axo00787-r	KIF23	axo02588-r	GCC2	axo02809-f	EIF4G1	axo05212-f	ACYP1
axo00518-f	DEDD2	axo00814-f	DAB2IP	axo02656-r	GLDN	axo02912-f	SASS6	axo05333-r	RP11-544M22.[illegible]
axo00622-r	ZNF418	axo00818-f	BCL2L14	axo02697-f	OLFML2A	axo03304-f	ZNF644	axo05395-f	MVK
axo00679-f	JOSD2	axo00946-f	ADCY4	axo03170-f	PRICKLE2	axo03376-f	RBBP8	axo05430-f	ADFP
axo00746-r	ADAT3	axo01302-f	C3orf75	axo03196-f	PTHLH	axo03512-r	RNF216	axo05779-r	CYBB
axo00766-r	ARL11	axo01345-f	ACOT11	axo03584-r	SGCG	axo03515-r	EXT2	axo05851-f	THAP5
axo00772-r	ZMYND19	axo01469-f	C1orf131	axo03840-f	Zfp316	axo03543-f	CLEC9A	axo05926-f	HYOU1
axo00797-r	BCL2L1	axo01531-r	LYSMD4	axo04307-f	CAST	axo04242-f	MTMR12	axo05927-r	HYOU1
axo00809-f	PPP1R14B	axo01543-f	TMEM56	axo04611-r	LAMA2	axo04262-r	PPAN-P2RY11	axo05978-f	RUNDC3B
axo00847-r	CANT1	axo01564-r	SNRNP48	axo05232-f	LAMA4	axo04392-f	PVRL2	axo06251-f	FDPS
axo00947-f	EHD4	axo01586-r	C10orf72	axo06235-r	TP53INP1	axo04451-f	ALAS2	axo06252-f	FDPS
axo00986-f	NEXN	axo01614-f	RALGPS2	axo06236-r	TP53INP1	axo04456-f	TOP2A	axo06329-f	LITAF
axo01025-f	TMEM182	axo01624-f	SLC10A4	axo06328-f	LITAF	axo04458-f	TOP2B	axo06723-f	SLC1A5
axo01163-f	ENKUR	axo01635-f	SAMD9L	axo06449-f	LUZP1	axo04482-f	QSER1	axo06790-f	LSS
axo01256-r	OSR1	axo01694-f	D2HGDH	axo06469-r	APRT	axo04578-r	KTN1	axo06807-f	LDLR
axo01322-r	HMGA1	axo01727-r	CPNE1	axo06546-r	BDNF	axo05082-r	RHAG	axo06808-f	LDLR
axo01328-r	CTSL1	axo01786-r	BRWD3	axo06627-r	DST	axo05388-f	NBEAL1	axo06921-f	KDM3A
axo01339-f	BVES	axo01930-f	ZNF398	axo06739-f	IKBKG	axo05737-r	SRRT	axo07323-f	B4GALT1

axo01342-f	SGMS1	axo01931-f	ZNF398	axo06842-f	PROM1	axo05886-f	DNMT1	axo07745-f	E2F3
axo01343-f	SGMS1	axo01953-r	USP2	axo07029-r	SERPINH1	axo06057-r	RTL1	axo07832-r	FRK
axo01351-f	FAM110B	axo01987-f	EYA2	axo07067-f	CHD4	axo06449-f	LUZP1	axo07927-f	HMGCS1
axo01403-f	RPUSD2	axo01993-f	KCNAB1	axo07124-r	CTSL2	axo06704-f	HBA2	axo07928-f	HMGCS1
axo01410-r	PARS2	axo02299-f	PCSK9	axo07205-f	IL1B	axo06705-f	HBA2	axo08089-r	LGALS9
axo01421-f	OBFC2A	axo02320-f	P2RX5	axo07255-f	EP300	axo06706-f	HBA2	axo08723-f	CYP51A1
axo01462-f	DDX59	axo02401-f	PPFIA1	axo07527-f	BHMT	axo07069-r	CHGA	axo08724-r	CYP51A1
axo01503-f	ZNF572	axo02443-f	TUBB2B	axo07643-f	COL4A1	axo07175-f	DYNC1H1	axo08747-f	DCK
axo01556-f	C3orf21	axo02494-r	Unknown	axo07645-f	COL4A2	axo07255-f	EP300	axo08748-f	DCK
axo01572-r	RASEF	axo02501-f	R3HDML	axo07646-f	COL6A1	axo07375-f	CYR61	axo08854-f	SPR
axo01587-f	ZNF567	axo02611-f	UPP1	axo07648-r	COL6A2	axo07737-f	DSG2	axo08863-f	SQLE
axo01603-f	TCEANC	axo02753-f	BPTF	axo07672-f	CPA3	axo07756-f	EGR1	axo08986-f	TK1
axo01680-f	LRRC43	axo02759-r	PCNA	axo07707-r	CTSG	axo07891-r	GOLGA4	axo09537-f	RIOK3
axo01743-f	APCDD1	axo02762-f	UBE2E1	axo07781-f	ADH4	axo07892-r	GOLGA4	axo09742-r	CDKN1B
axo01745-f	ZFP3	axo02772-f	ANKDD1A	axo07891-r	GOLGA4	axo07983-f	ITGA2	axo09755-f	DDIT3
axo01779-r	TMEM92	axo02775-f	CREM	axo08060-f	LAMB1	axo08016-f	JUNB	axo10093-r	ACSL4
axo01795-f	COL29A1	axo02805-f	SYNE2	axo08427-r	PNN	axo08253-f	TSPO	axo10556-f	ATP6V1G1
axo01878-f	ZNF367	axo02809-f	EIF4G1	axo08445-f	PPL	axo08341-f	PARN	axo10822-f	FGFBP1
axo01897-f	PHF13	axo02837-f	ZMYND8	axo08501-f	HTRA1	axo08585-r	PTPRG	axo11001-f	HSP90AA1
axo01906-f	H1FOO	axo02839-r	ZMYND8	axo08599-f	PTX3	axo08713-f	CLIP1	axo11173-f	INSIG1
axo01935-f	TGIF1	axo02912-f	SASS6	axo08812-f	SMARCC1	axo08941-f	TCF3	axo11277-f	PSCA
axo01936-f	LMNA	axo02937-f	LBR	axo08860-r	SPTBN1	axo09180-f	USP7	axo11428-f	SLC35B1
axo01937-f	LMNA	axo02972-f	SCML4	axo09009-f	CLEC3B	axo09186-f	CUL5	axo11646-f	LIPG
axo01943-r	RASSF1	axo02994-f	GRHL1	axo09304-f	CILP	axo09271-f	NSMAF	axo11879-f	DNAJC3
axo01953-r	USP2	axo03039-f	CMIP	axo09522-f	TNFSF10	axo09319-f	PPFIBP1	axo12025-f	DDX17
axo02043-f	NR4A1	axo03052-r	APOOL	axo09717-f	ANXA6	axo09451-f	EIF3A	axo12197-f	PMVK
axo02050-r	KCNIP2	axo03089-f	KIT	axo09985-f	CCNG2	axo09985-f	CCNG2	axo12280-f	MTHFD2
axo02051-f	NR4A3	axo03098-f	TBC1D10C	axo09986-f	CCNG2	axo10049-r	DSP	axo12378-f	SC4MOL
axo02052-f	TGIF1	axo03123-f	GJB7	axo10048-f	DSP	axo10093-r	ACSL4	axo12512-f	KDELR3
axo02069-r	MCAT	axo03126-r	C7orf52	axo10063-r	EFNA1	axo10523-f	CLTC	axo13132-r	DNAJB9
axo02097-r	GLUL	axo03136-f	SULF2	axo10102-r	FAP	axo10526-r	CLTC	axo13165-r	NUPR1
axo02098-f	GLUL	axo03266-r	KRT12	axo10242-f	PTGS1	axo10775-r	ISG15	axo14255-f	KIAA0831
axo02163-f	RNF149	axo03304-f	ZNF644	axo10279-f	RARG	axo10931-f	FOS	axo15168-f	WSB1
axo02215-f	HARBI1	axo03347-r	CDC25A	axo10523-f	CLTC	axo11001-f	HSP90AA1	axo15758-r	ISYNA1
axo02271-f	NDUFAF2	axo03362-r	TGM5	axo10526-r	CLTC	axo11120-f	SMC4	axo15946-r	RWDD2B
axo02274-f	C14orf79	axo03391-f	SHC4	axo10713-f	RELN	axo11122-f	SMC4	axo16986-r	ETNK1

axo02299-f	PCSK9
axo02347-f	SMOX
axo02356-f	ZNF792
axo02359-f	FLJ36031
axo02391-f	PPAP2A
axo02394-f	MIDN
axo02413-f	UNC45B
axo02443-f	TUBB2B
axo02459-r	P2RY8
axo02611-f	UPP1
axo02695-f	CPEB2
axo02704-f	ZFAND2A
axo02731-f	BCL9L
axo02775-f	CREM
axo02776-f	TXNRD1
axo02795-f	FILIP1L
axo02823-f	IRF2BP2
axo02833-r	DLGAP4
axo02870-r	C6orf145
axo02877-f	RNF14
axo02906-r	ARL4A
axo03042-r	FLRT3
axo03054-f	PDE4B
axo03082-r	SMTNL2
axo03098-f	TBC1D10C
axo03196-f	PTHLH
axo03234-r	ADSSL1
axo03238-f	PMEPA1
axo03262-f	SPRY1
axo03268-f	KRT12
axo03338-r	CDC42SE2
axo03373-r	RP9
axo03393-f	PDLIM7
axo03405-f	FITM1
axo03408-f	RCAN1
axo03393-f	PDLIM7
axo03457-f	RASL11A
axo03472-f	BACH1
axo03528-f	MBNL2
axo03702-f	LOC731511
axo04192-f	CD68
axo04208-f	PEG10
axo04213-r	PEG10
axo04266-f	INCENP
axo04267-r	INCENP
axo04273-f	MPO
axo04294-f	MSH2
axo04349-r	TATDN3
axo04396-f	STAG2
axo04448-f	TCN1
axo04520-f	NCOR2
axo04544-f	LRRCC1
axo04567-r	CUBN
axo04584-f	TRAPPC6B
axo04603-f	INSR
axo04649-r	KIAA1147
axo04741-r	CLEC2L
axo04906-r	PON2
axo04909-r	GPR34
axo05068-f	CYP4B1
axo05106-f	RLBP1
axo05160-f	hCG_1645727
axo05213-f	FAM70A
axo05303-f	ACP5
axo05513-f	ARG1
axo05517-f	CEP55
axo05524-f	TET2
axo05682-f	WDSUB1
axo05712-r	SMARCAD1
axo05722-r	ELFN1
axo11122-f	SMC4
axo11164-f	HSPG2
axo11207-r	LOXL1
axo11338-r	HDGF2
axo11431-r	CALCOCO2
axo11497-f	MAP1B
axo12036-f	USP16
axo12055-r	GPM6B
axo12171-f	ZNF217
axo12218-f	AVIL
axo12273-r	ZMYND11
axo12885-f	MMRN1
axo13184-f	PLXNB2
axo13429-f	DHX38
axo13709-f	SACS
axo13919-f	ZNF592
axo13920-f	ZNF592
axo13970-r	CLINT1
axo14975-f	NIPBL
axo15011-r	ABI3BP
axo15275-f	TRIM72
axo15286-f	LMO7
axo15621-f	PACSIN3
axo15713-f	RHCG
axo15825-r	NT5C3
axo16085-f	ZCCHC8
axo16106-f	TET2
axo16121-f	KIF21A
axo16929-f	C6orf35
axo17277-f	ITSN2
axo17755-f	ARRDC3
axo18098-f	SCAF1
axo18172-f	ADAM28
axo18390-r	MLF1
axo18492-f	NUCKS1
axo11224-r	ATP8B1
axo11241-f	SGK1
axo11497-f	MAP1B
axo11802-r	OAS3
axo11934-r	VARS
axo12036-f	USP16
axo12171-f	ZNF217
axo12273-r	ZMYND11
axo12607-f	WASF2
axo12724-f	VEZF1
axo13033-f	PRDM2
axo13076-f	TLK1
axo13121-f	LETM1
axo13419-f	MYOF
axo13520-f	SETD2
axo13600-f	SLC25A15
axo13759-f	AFF4
axo14041-f	SLK
axo14042-f	SLK
axo14063-f	MTSS1
axo14354-r	SPEN
axo14365-f	SPEN
axo14424-f	PDS5B
axo14779-f	KIAA0664
axo14975-f	NIPBL
axo15025-f	AHCTF1
axo15286-f	LMO7
axo15433-r	FBN1
axo15466-f	FAHD2A
axo15676-f	TAF9
axo15693-f	GPN3
axo15906-r	UFM1
axo16166-r	GIN1
axo16391-f	HERC6
axo16897-r	CHST11
axo17115-f	SLC38A2
axo17249-f	GTPBP2
axo18258-f	SDF2L1
axo18590-r	C12orf43
axo18653-f	AACS
axo18712-r	DCTPP1
axo18752-r	CRELD2
axo18950-f	E2F8
axo18964-r	MUC16
axo21889-r	Unknown
axo23008-f	LOC100145494
axo23514-f	Pdcd1lg2
axo23651-f	PNAT3
axo24047-f	Prod 1
axo24396-f	pepE
axo24544-f	Fdft1
axo25777-f	Unknown
axo27383-f	Unknown
axo27913-f	Unknown
axo28223-f	Unknown
axo28231-f	Unknown
axo29829-f	Unknown
axo29854-f	Unknown
axo30518-f	Unknown
axo30940-f	Unknown
axo31339-f	Unknown
axo31341-f	Unknown
axo31453-f	Unknown
axo31546-f	Unknown
axo31671-f	Unknown

axo03422-f	LEP	axo05737-r	SRRT	axo18655-f	ZBTB10	axo16984-f	DNAJA4
axo03429-f	KEAP1	axo05826-f	VWA5A	axo18882-f	PLEKHF2	axo17147-r	FAM190B
axo03457-f	RASL11A	axo05870-f	PPP3CA	axo19257-f	COQ10B	axo17755-f	ARRDC3
axo03514-r	LRRC20	axo05917-f	PLCH1	axo19270-r	ELL3	axo18172-f	ADAM28
axo03584-r	SGCG	axo05927-r	HYOU1	axo19273-f	NINL	axo18366-f	ZFYVE20
axo03610-f	ZNF543	axo05962-f	IFNGR1	axo19600-f	CCDC3	axo18601-f	ZNF638
axo03630-f	CHCHD10	axo05977-f	IL4R	axo19812-f	SLITRK6	axo18882-f	PLEKHF2
axo03686-f	LOC10013359[illegible]	axo05984-r	CDC7	axo19969-f	PHF6	axo18986-f	GRTP1
axo03758-f	FLJ22184	axo05994-f	DSG4	axo20034-f	CARD6	axo18989-f	ZFHX4
axo03790-r	Zfp35	axo06032-f	KRT5	axo20282-r	RHPN2	axo19229-f	MUS81
axo03836-f	LOC10013106[illegible]	axo06046-r	RTL1	axo21577-f	Unknown	axo19257-f	COQ10B
axo03843-f	LOC285095	axo06109-r	RTL1	axo21650-r	LOC496300	axo19273-f	NINL
axo03985-r	YIF1B	axo06111-r	RTL1	axo22289-f	MGC145244	axo19554-f	EPPK1
axo04102-f	LOC643596	axo06120-r	RTL1	axo23396-f	zgc:194655	axo19556-f	EPPK1
axo04163-f	AIM1L	axo06163-f	ASPA	axo23458-r	Unknown	axo19782-f	NR3C1
axo04224-f	LYZ	axo06179-f	TCOF1	axo23595-f	BIK	axo20128-f	SH3KBP1
axo04273-f	MPO	axo06203-r	ARHGAP26	axo23816-f	Unknown	axo20130-f	SH3KBP1
axo04404-f	TPM3	axo06248-r	TMEM2	axo24112-r	Lynx1	axo20165-r	HBG2
axo04424-f	SSTR2	axo06262-f	MYOT	axo24424-f	LOC10022866[illegible]	axo20166-r	HBG2
axo04425-f	SLC7A3	axo06349-r	LRRFIP1	axo24990-f	Unknown	axo20399-f	HBD
axo04448-f	TCN1	axo06448-f	SLC35B3	axo27674-f	Unknown	axo21577-f	Unknown
axo04451-f	ALAS2	axo06551-f	PLEKHA5	axo27795-f	Unknown	axo21650-r	LOC496300
axo04452-f	NCF1	axo06563-r	NOX4	axo27847-f	Unknown	axo21847-f	Unknown
axo04581-f	CYP4F2	axo06612-f	NHSL1	axo27866-f	Unknown	axo21930-r	Unknown
axo04606-f	ITGA6	axo06619-f	CHORDC1	axo28320-f	Unknown	axo22034-f	Unknown
axo04621-f	GYG2	axo06715-f	FAM190A	axo28383-f	Unknown	axo22270-f	Unknown
axo04639-f	LDB3	axo06717-r	SKIL	axo28704-f	Unknown	axo22523-r	Unknown
axo04649-r	KIAA1147	axo06727-r	FLJ25076	axo29695-f	Unknown	axo23122-f	RREB1
axo04651-f	GLT8D4	axo06840-f	ROBO1	axo29708-f	Unknown	axo23215-f	ticrr
axo04687-r	TTC36	axo06883-f	NEK3	axo29831-f	Unknown	axo23458-r	Unknown
axo04765-f	SPI1	axo06891-f	PTGR2	axo29905-f	Unknown	axo23508-f	LOC100182771
axo04865-f	MRAS	axo06906-f	AIFM3	axo30536-f	Unknown	axo23600-f	BEST1
axo04910-r	RPP38	axo06988-r	ARHGDIB	axo31329-f	Unknown	axo23749-f	pycard
axo05160-f	hCG_1645727	axo06993-f	ATR	axo31357-f	Unknown	axo23816-f	Unknown
axo05181-r	RP5-1000E10.[illegible]	axo07032-f	CCNA2	axo31395-f	Unknown	axo24313-f	Unknown
axo05184-r	RP5-1000E10.[illegible]	axo07046-r	CDC5L	axo31404-f	Unknown	axo24400-r	Unknown

axo05187-f	ZFAND5
axo05194-r	SNX18
axo05214-f	ELOVL7
axo05224-f	SLC25A36
axo05228-f	TLE3
axo05267-f	UGT2A2
axo05289-f	GJB6
axo05307-f	EZR
axo05328-f	BRD2
axo05362-f	TGM1
axo05430-f	ADFP
axo05451-f	MYOZ3
axo05453-f	TYR
axo05497-f	LNX1
axo05513-f	ARG1
axo05531-f	ACCS
axo05637-f	PRG4
axo05645-r	MMP2
axo05697-f	C13orf31
axo05735-f	RRAD
axo05779-r	CYBB
axo05801-f	MOV10
axo05844-r	SSFA2
axo05901-f	ENPP2
axo05907-r	ERVWE1
axo05997-f	KIAA0182
axo06005-r	PLCXD2
axo06014-r	KRT17
axo06142-f	MUC15
axo06225-f	PENK
axo06235-r	TP53INP1
axo06236-r	TP53INP1
axo06337-f	ALOX12B
axo06349-r	LRRFIP1
axo06372-r	FGF13
axo06406-r	TXNL4B
axo07047-f	CDC6
axo07127-f	CTSZ
axo07183-f	BLM
axo07299-f	FLT3LG
axo07375-f	CYR61
axo07482-r	RHOA
axo07495-f	ATP1B2
axo07527-f	BHMT
axo07530-f	BNC1
axo07584-r	IL8RA
axo07616-f	CTSC
axo07626-f	CKS1B
axo07684-f	CRYAB
axo07697-f	CTGF
axo07739-r	HBEGF
axo07788-f	F3
axo07930-r	RBMX
axo07980-r	IRF2
axo07993-r	ITGB5
axo08031-r	ALOX12
axo08060-f	LAMB1
axo08130-f	MAD2L1
axo08154-f	MCM3
axo08253-f	TSPO
axo08258-f	NASP
axo08259-f	NASP
axo08386-r	CALCB
axo08408-f	PKM2
axo08429-r	POLA2
axo08436-f	POLE2
axo08501-f	HTRA1
axo08535-r	PSMD1
axo08550-f	PTBP1
axo08653-f	RBL1
axo08726-f	MAPK12
axo08748-f	DCK
axo31535-f	Unknown
axo31574-f	Unknown
axo31607-f	Unknown
axo24446-r	Unknown
axo24493-f	Unknown
axo25463-f	Unknown
axo26284-f	Unknown
axo27248-f	Unknown
axo28076-f	Unknown
axo29345-f	Unknown
axo29410-f	Unknown
axo29664-f	Unknown
axo29987-f	Unknown
axo30920-f	Unknown
axo31329-f	Unknown
axo31429-f	Unknown
axo31669-f	Unknown

axo06411-f	CMKLR1	axo08772-f	SH3GL2
axo06445-f	FBXO5	axo08776-f	ST8SIA1
axo06498-r	DLG2	axo08791-r	SLC16A1
axo06518-r	COL17A1	axo08801-r	SMARCA2
axo06546-r	BDNF	axo08802-r	SMARCA2
axo06589-f	WEE1	axo08814-r	SMARCE1
axo06600-f	SEC14L1	axo08834-f	SOAT1
axo06608-r	FBP1	axo08835-f	SOD3
axo06643-f	CCNL2	axo08986-f	TK1
axo06656-f	PRICKLE1	axo09019-r	TPM4
axo06661-f	CLEC4M	axo09088-f	USP4
axo06665-r	MX1	axo09108-f	VIM
axo06710-f	ZNF286B	axo09110-f	VIM
axo06723-f	SLC1A5	axo09166-r	ZNF143
axo06725-r	NR2F2	axo09176-f	CHIT1
axo06727-r	FLJ25076	axo09178-f	SCG2
axo06768-r	ZBTB22	axo09180-f	USP7
axo06773-f	OTUD1	axo09186-f	CUL5
axo06804-r	BCHE	axo09206-r	TRRAP
axo06805-r	BCHE	axo09290-f	SMARCA5
axo06807-f	LDLR	axo09318-f	PPFIBP2
axo06808-f	LDLR	axo09449-f	EIF3A
axo06854-f	MMP1	axo09451-f	EIF3A
axo07001-f	BMP2	axo09551-f	SUCLG2
axo07042-r	CCNE1	axo09628-f	PRPF4B
axo07047-f	CDC6	axo09781-r	FEN1
axo07074-f	CIRBP	axo09801-f	MYO9B
axo07098-f	CLDN4	axo09808-f	ORC1L
axo07100-f	CLDN7	axo09859-f	PTTG1
axo07131-f	CD53	axo09986-f	CCNG2
axo07205-f	IL1B	axo09994-f	CDH1
axo07206-r	IL1B	axo10019-f	VCAN
axo07246-f	EMP1	axo10048-f	DSP
axo07260-f	EREG	axo10057-f	ECM1
axo07288-f	FOXC1	axo10082-f	EPRS
axo07301-r	FZD2	axo10086-f	EPS8

axo07306-r	IL8
axo07311-f	GATM
axo07346-f	HAS1
axo07368-f	IGSF3
axo07375-f	CYR61
axo07395-f	SERPINE1
axo07403-r	ACTA2
axo07436-f	AIF1
axo07465-f	AQP4
axo07473-f	AREG
axo07477-f	ARL4D
axo07479-f	ARF6
axo07527-f	BHMT
axo07547-f	BTG1
axo07584-r	IL8RA
axo07591-f	CDH2
axo07673-f	TGFB1
axo07684-f	CRYAB
axo07719-f	GADD45A
axo07739-r	HBEGF
axo07740-f	DUSP7
axo07756-f	EGR1
axo07788-f	F3
axo07820-f	FOXO1
axo07893-f	GOT1
axo07929-r	HMOX1
axo07935-r	HNRNPK
axo07944-f	HSD17B2
axo07949-f	TNC
axo07965-f	ID1
axo08016-f	JUNB
axo08027-r	KCNJ13
axo08028-f	KCNS3
axo08070-f	LCP1
axo08089-r	LGALS9
axo08095-r	LMX1B
axo10365-r	RABEP1
axo10398-f	COL3A1
axo10419-r	DHRS3
axo10425-f	COPB2
axo10444-f	SLIT2
axo10502-f	AKAP7
axo10539-f	VPS4B
axo10608-f	DNASE1L3
axo10620-f	FUS
axo10695-f	SERPINB10
axo10761-f	PGLYRP1
axo10788-r	COL7A1
axo10800-r	HS3ST1
axo10867-r	CAPN1
axo10885-f	CSF1R
axo10953-r	GPR4
axo10968-f	HIST1H1B
axo11000-f	HSPA5
axo11001-f	HSP90AA1
axo11087-f	CHAF1B
axo11111-r	CHAF1A
axo11112-f	CHAF1A
axo11113-f	CHAF1A
axo11119-r	SMC4
axo11120-f	SMC4
axo11122-f	SMC4
axo11193-r	LGALS3BP
axo11205-f	LMNB1
axo11219-r	NIT1
axo11387-f	RBM5
axo11484-f	CD5L
axo11498-f	MAP1B
axo11511-r	MCM4
axo11512-f	MCM4
axo11514-r	MCM6
axo11515-f	MCM7

axo08122-f	EPCAM	axo11566-f	MYH10
axo08123-f	EPCAM	axo11580-r	HNRNPM
axo08144-f	MAS1	axo11612-r	TUBA1A
axo08148-r	MATN2	axo11699-r	TUBB2C
axo08153-f	MCM3	axo11718-f	WFDC2
axo08156-r	MDM2	axo11777-f	NFE2
axo08174-f	MMP1	axo11926-f	TLN1
axo08175-f	MMP1	axo11975-r	RBM14
axo08178-f	MMP3	axo12021-r	PAMR1
axo08179-f	MMP3	axo12025-f	DDX17
axo08180-f	MMP3	axo12061-f	OLFM4
axo08181-f	MMP3	axo12092-r	DRAP1
axo08183-f	MMP8	axo12095-r	SMC2
axo08184-f	MMP10	axo12097-f	SMC2
axo08186-f	MMP13	axo12121-f	IVNS1ABP
axo08187-f	MMP19	axo12136-r	RAD51AP1
axo08195-f	MRC1	axo12248-f	PTGES3
axo08200-f	MRC1	axo12280-f	MTHFD2
axo08201-f	MSH3	axo12320-f	FGL2
axo08256-f	MYOG	axo12374-f	MCM5
axo08282-f	NGFR	axo12375-f	RBP4
axo08309-f	ODC1	axo12401-f	MARCO
axo08386-r	CALCB	axo12424-f	EHD1
axo08387-r	PGAM1	axo12442-f	CBX1
axo08399-f	CCK	axo12504-f	KIF2C
axo08403-f	PIM1	axo12522-f	STON1
axo08467-f	PRG2	axo12641-r	TOPBP1
axo08487-r	MAPK6	axo12693-f	TFDP1
axo08505-f	PRTN3	axo12701-f	TMF1
axo08614-f	CYP2C8	axo12802-r	PACSIN2
axo08667-f	RDX	axo12856-f	BRCA1
axo08686-r	RGS2	axo12882-f	ATF6
axo08731-f	SAT1	axo12914-f	TARDBP
axo08744-f	SDC4	axo12922-f	CCT5
axo08767-f	SFTPD	axo13106-f	EPB41L3
axo08791-r	SLC16A1	axo13108-f	EPB41L3

axo08844-f	UAP1	axo13110-f	EPB41L3
axo08845-f	UAP1	axo13117-f	KIF4A
axo08897-f	STX5	axo13240-f	TLL1
axo08965-f	TGM3	axo13266-f	STK39
axo08967-f	THBS1	axo13318-f	UHRF1
axo08979-f	TIMP1	axo13418-f	MYOF
axo09045-f	PHLDA2	axo13500-f	ATAD2
axo09064-f	UBA1	axo13513-f	WDR91
axo09083-r	UGCG	axo13557-f	PKP1
axo09084-f	UGDH	axo13581-r	GPNMB
axo09106-r	VGF	axo13659-f	HEBP2
axo09113-f	VLDLR	axo13660-f	HEBP2
axo09128-f	ZFP36	axo13661-f	ORC6L
axo09141-f	ZNF268	axo13683-f	ZNF318
axo09156-r	ZNF135	axo13685-f	ZNF318
axo09175-r	CHIT1	axo13686-f	ZNF318
axo09176-f	CHIT1	axo13688-f	ZNF318
axo09177-f	CXCR4	axo13689-f	ZNF318
axo09178-f	SCG2	axo13776-r	PKP4
axo09216-f	GSTP1	axo13846-f	TFCP2L1
axo09402-r	SLC25A12	axo13895-f	CYFIP1
axo09440-f	SOCS1	axo14121-f	KIAA0528
axo09521-f	TNFSF10	axo14239-r	VASH1
axo09537-f	RIOK3	axo14279-f	ANKRD6
axo09566-f	CES2	axo14306-f	PDCD11
axo09606-f	SQSTM1	axo14365-f	SPEN
axo09691-f	MYOM2	axo14494-f	PEG10
axo09704-f	CLK2	axo14531-f	ZC3H13
axo09745-f	PLK3	axo14534-f	ZC3H13
axo09755-f	DDIT3	axo14726-r	DNAJC9
axo09771-r	F2RL2	axo14784-f	KIAA0664
axo09862-f	STK17B	axo14824-r	NCAPD3
axo09873-f	KLF4	axo14857-r	FABP2
axo09981-f	CAMP	axo14901-f	KIAA0776
axo09996-f	CEACAM5	axo14971-f	NIPBL
axo10051-f	DUSP1	axo14972-f	NIPBL

axo10052-f	DUSP5	axo14985-r	DKFZP564O0823
axo10093-r	ACSL4	axo15166-f	CCDC69
axo10221-f	YBX1	axo15284-f	FCN2
axo10259-f	PTGS2	axo15289-r	KRT76
axo10260-f	PTGS2	axo15390-r	THAP4
axo10275-f	TSPAN7	axo15466-f	FAHD2A
axo10319-f	RAB11A	axo15540-f	IRAK4
axo10353-f	SLC16A6	axo15553-f	MYEF2
axo10354-f	SLC16A6	axo15588-f	CERCAM
axo10356-f	SLC16A6	axo15621-f	PACSIN3
axo10367-f	PRKRIR	axo15633-r	GPRC5B
axo10458-f	ADIPOQ	axo15688-f	PCYOX1
axo10555-f	CXCL14	axo15755-f	DSP
axo10587-f	C21orf2	axo15875-f	TLR7
axo10655-f	ABCC1	axo15956-f	USP18
axo10676-f	NRCAM	axo15971-f	BCMO1
axo10743-f	XBP1	axo15982-f	AZI1
axo10749-f	PLA2G7	axo16047-f	PARP14
axo10761-f	PGLYRP1	axo16088-f	DONSON
axo10775-r	ISG15	axo16125-f	HAUS6
axo10795-f	GFPT2	axo16239-r	NCAPG2
axo10796-r	GFPT2	axo16297-f	EXD3
axo10800-r	HS3ST1	axo16386-r	C11orf59
axo10822-f	FGFBP1	axo16459-f	FAM83B
axo10855-r	RND3	axo16479-r	SOBP
axo10910-f	ETS2	axo16578-f	CDCA8
axo10912-f	F2RL1	axo16582-f	DOK4
axo10931-f	FOS	axo16703-f	LRRC1
axo10934-f	GAK	axo16732-r	KIRREL
axo10994-r	NCKAP1L	axo16744-r	CDK5RAP2
axo11000-f	HSPA5	axo16896-f	CHST11
axo11001-f	HSP90AA1	axo16976-r	MCM10
axo11003-r	JUND	axo16984-f	DNAJA4
axo11014-r	MYCN	axo17017-f	MLL5
axo11025-f	NFIL3	axo17025-r	ANLN
axo11037-f	PPP1R3C	axo17075-r	PCDHGA10

axo11039-f	PRKAB2	axo17173-f	HEATR5B
axo11058-f	SLC20A1	axo17184-f	RHOF
axo11082-f	MALL	axo17210-f	EXOC6
axo11152-f	CALHM1	axo17340-f	UGGT1
axo11205-f	LMNB1	axo17395-f	ENY2
axo11233-f	DLL1	axo17495-f	NKIRAS1
axo11241-f	SGK1	axo17641-r	LRRC47
axo11262-f	KLF10	axo17772-f	VPS13C
axo11274-f	ST8SIA4	axo17826-f	LRRN1
axo11320-f	TSPAN3	axo17865-f	FXR1
axo11372-f	LPAR6	axo18008-f	PLSCR1
axo11392-f	TNK2	axo18021-f	POLR2L
axo11424-f	M6PRBP1	axo18040-f	ANGPTL7
axo11443-r	IGSF6	axo18165-r	MAD1L1
axo11504-r	MAT2A	axo18248-r	ZNF711
axo11505-f	MAT2A	axo18274-r	FAM38B
axo11576-f	NAB1	axo18486-r	ELOVL4
axo11646-f	LIPG	axo18492-f	NUCKS1
axo11684-r	CITED2	axo18510-f	TNS3
axo11685-f	CITED2	axo18539-f	CERK
axo11748-f	CYBA	axo18619-f	KRI1
axo11749-r	PIM3	axo18772-f	DSC3
axo11757-f	DNAJB1	axo18773-f	ATP10A
axo11777-f	NFE2	axo18779-f	GLDC
axo11780-f	NNMT	axo18816-f	ADIPOR2
axo11784-r	NNMT	axo18855-f	MAP7D3
axo11798-f	NR4A2	axo18905-r	MAK10
axo12061-f	OLFM4	axo18906-f	MAEA
axo12103-f	CDC42EP3	axo18940-f	ESCO2
axo12159-f	SLC2A1	axo19105-f	GRHL2
axo12161-f	SLC2A1	axo19121-f	ESRP2
axo12174-f	TFPI2	axo19297-f	PLEKHO2
axo12175-f	TFPI2	axo19319-f	RAB28
axo12219-r	B3GNT2	axo19366-r	DDHD1
axo12320-f	FGL2	axo19367-f	DUSP16
axo12321-f	FGL2	axo19375-f	SH3PXD2B

axo12335-f	GADD45G	axo19378-f	DDX11
axo12350-f	MCTP1	axo19386-f	SERPINB1
axo12364-r	HIVEP2	axo19445-r	TPM1
axo12380-f	SLC20A2	axo19472-f	KAZALD1
axo12424-f	EHD1	axo19486-f	LOC81691
axo12439-f	BTG3	axo19506-f	HNRNPA1
axo12526-r	GMPR	axo19509-f	MSH6
axo12531-f	ZFP36L2	axo19512-f	HYI
axo12561-r	SILV	axo19615-f	TRPT1
axo12565-f	SMTN	axo19652-f	SYT16
axo12599-f	MTERF	axo19749-f	CEP192
axo12601-f	ALX1	axo19806-f	ZCCHC7
axo12605-f	ADAMTS1	axo19827-r	ZNRF1
axo12724-f	VEZF1	axo19914-f	ARF1
axo12734-f	ZXDB	axo19923-r	BAZ1B
axo12755-f	PKP3	axo19925-f	BAZ1B
axo12925-f	ELL2	axo19944-f	EFCAB7
axo12926-r	ELL2	axo19967-f	PCDH7
axo12982-f	LPAR3	axo20022-f	DGAT2
axo12999-f	FBXO5	axo20065-r	CNDP1
axo13004-f	DUPD1	axo20102-f	ZC3H10
axo13049-r	RHOQ	axo20184-f	ALKBH6
axo13132-r	DNAJB9	axo20287-r	PNPT1
axo13133-f	MMD	axo20305-r	CALD1
axo13185-f	PLXNB2	axo20609-f	RBMX
axo13216-r	SEMA3E	axo20630-f	NASP
axo13259-r	ACAN	axo20743-f	FABP3
axo13263-f	FLRT2	axo20840-f	SEPP1
axo13289-r	ZNF214	axo20953-f	EBNA1BP2
axo13297-r	ZNF180	axo21055-f	TRAPPC2
axo13324-f	RBM15B	axo21059-f	KIAA0101
axo13352-r	SNX10	axo21474-f	RAB7B
axo13398-r	FTSJ2	axo21578-f	LOC580034
axo13423-f	NRG1	axo21644-f	Unknown
axo13472-f	ACAD9	axo21675-f	Unknown
axo13532-f	PHPT1	axo21717-f	acbd5

axo13670-f	SLC7A11	axo21741-f	LOC100158321
axo13692-f	TNFAIP8	axo22170-f	Unknown
axo13778-f	TNFRSF21	axo22179-f	CATHL3
axo13780-f	SESN1	axo22195-f	Unknown
axo13790-r	SULT1B1	axo22371-r	CATHL3
axo13793-f	TEKT2	axo22729-f	Unknown
axo13846-f	TFCP2L1	axo22772-f	Unknown
axo13847-f	TFCP2L1	axo22852-r	Unknown
axo13864-f	LMCD1	axo22914-r	Unknown
axo13866-f	ERO1L	axo22982-r	Unknown
axo13904-r	PSME4	axo23137-f	LOC100217844
axo13990-f	HERPUD1	axo23215-f	ticrr
axo14052-r	KIAA0247	axo23458-r	Unknown
axo14096-r	TSC22D2	axo23488-f	LOC100182771
axo14109-r	ZEB2	axo23508-f	LOC100182771
axo14150-f	ZC3H11A	axo23595-f	BIK
axo14242-r	ADNP2	axo23596-f	LOC100228006
axo14253-f	CHSY1	axo23816-f	Unknown
axo14260-f	ZNF510	axo23885-f	Unknown
axo14342-f	SPEN	axo23962-f	Unknown
axo14380-f	TMCC1	axo23974-f	CCL19
axo14439-f	SDC1	axo24309-f	ctnnal1
axo14488-f	PPRC1	axo24428-f	LOC100230619
axo14631-f	ZNF182	axo24612-f	Unknown
axo14810-f	ATMIN	axo24666-f	Unknown
axo14898-r	ARHGEF4	axo24811-f	Unknown
axo15011-r	ABI3BP	axo24936-f	Unknown
axo15057-f	NIPSNAP3A	axo24960-f	Unknown
axo15069-r	KBTBD2	axo24990-f	Unknown
axo15086-r	TIPARP	axo25215-f	Unknown
axo15168-f	WSB1	axo25241-f	Unknown
axo15169-f	WSB1	axo25303-f	Unknown
axo15260-f	FAH	axo25360-f	Unknown
axo15268-f	G0S2	axo25450-f	Unknown
axo15275-f	TRIM72	axo25513-f	Unknown
axo15307-f	PRLH	axo25542-f	Unknown

axo15328-r	GMNN	axo25564-f	Unknown
axo15359-r	LIPT1	axo25678-f	Unknown
axo15468-f	SLMO2	axo25710-f	Unknown
axo15633-r	GPRC5B	axo25735-f	Unknown
axo15663-f	KLF2	axo25775-f	Unknown
axo15793-f	PLEK2	axo25896-f	Unknown
axo15852-f	KLF3	axo25928-f	Unknown
axo15882-f	ECSIT	axo26017-f	Unknown
axo15900-f	SLC25A37	axo26383-f	Unknown
axo15915-f	TNFRSF12A	axo26502-f	Unknown
axo15921-f	DACT1	axo26821-f	Unknown
axo16020-f	MYH4	axo26905-f	Unknown
axo16068-f	CHRNA9	axo26930-f	Unknown
axo16074-f	DIRAS2	axo26988-f	Unknown
axo16109-f	FAM46A	axo27232-f	Unknown
axo16187-f	ANKRD16	axo27481-f	Unknown
axo16194-f	ANKRD49	axo27517-f	Unknown
axo16210-f	EPS8L1	axo27567-f	Unknown
axo16295-f	RG9MTD1	axo27672-f	Unknown
axo16297-f	EXD3	axo27796-f	Unknown
axo16299-f	RHBDL2	axo27891-f	Unknown
axo16300-f	C12orf41	axo28206-f	Unknown
axo16336-f	TMEM70	axo28211-f	Unknown
axo16389-r	TRMT61B	axo28223-f	Unknown
axo16391-f	HERC6	axo28253-f	Unknown
axo16475-f	ARGLU1	axo28336-f	Unknown
axo16477-f	KIF26B	axo28354-f	Unknown
axo16479-r	SOBP	axo28680-f	Unknown
axo16528-r	C16orf91	axo28765-f	Unknown
axo16630-f	RNF220	axo28879-f	Unknown
axo16641-r	DNAJC17	axo28950-f	Unknown
axo16783-f	ZYX	axo29182-f	Unknown
axo16843-r	LARP6	axo29193-f	Unknown
axo16867-f	FBXL8	axo29426-f	Unknown
axo16899-f	PDLIM5	axo29450-f	Unknown
axo16918-f	PDP1	axo29746-f	Unknown

axo16984-f	DNAJA4	axo29843-f	Unknown
axo17005-f	BATF3	axo29976-f	Unknown
axo17034-r	CHCHD7	axo30119-f	Unknown
axo17075-r	PCDHGA10	axo30246-f	Unknown
axo17084-f	TUBA8	axo30258-f	Unknown
axo17091-f	CYCS	axo30357-f	Unknown
axo17093-f	ERRFI1	axo30477-f	Unknown
axo17115-f	SLC38A2	axo30669-f	Unknown
axo17152-f	ETAA1	axo30681-f	Unknown
axo17172-r	CENPP	axo30728-f	Unknown
axo17185-f	RHOF	axo30782-f	Unknown
axo17186-r	PCDH18	axo30808-f	Unknown
axo17187-r	PCDH18	axo30895-f	Unknown
axo17219-f	DDIT4	axo30940-f	Unknown
axo17249-f	GTPBP2	axo31194-f	Unknown
axo17286-r	IRGC	axo31203-f	Unknown
axo17386-f	FEM1C	axo31209-f	Unknown
axo17387-f	PMEPA1	axo31329-f	Unknown
axo17396-f	OLFML3	axo31346-f	Unknown
axo17413-r	C5orf15	axo31364-f	Unknown
axo17422-f	RGMA	axo31404-f	Unknown
axo17430-r	PRDM9	axo31418-f	Unknown
axo17458-f	CCNL1	axo31458-f	Unknown
axo17461-f	CXCR7	axo31486-f	Unknown
axo17518-f	GPR84	axo31527-f	Unknown
axo17557-f	SALL4	axo31531-f	Unknown
axo17614-r	CLK4	axo31557-f	Unknown
axo17626-f	DSCAML1	axo31580-f	Unknown
axo17661-r	RIMKLB	axo31587-f	Unknown
axo17674-f	ZNF608	axo31602-f	Unknown
axo17780-f	LSP1	axo31608-f	Unknown
axo17810-r	SEMA6D	axo31613-f	Unknown
axo17840-r	ZNF530	axo31651-f	Unknown
axo17852-f	KIAA1524		
axo17888-r	ZBTB26		
axo17955-f	FAM53A		

axo17957-r	CCL3L1
axo17997-f	NMB
axo18055-f	GRHL3
axo18067-f	SLC30A1
axo18068-f	CLDN6
axo18074-f	RHOU
axo18086-f	OVOL2
axo18131-f	PELI2
axo18217-r	BCAN
axo18332-r	SAMSN1
axo18342-r	C10orf54
axo18365-f	RAB38
axo18410-f	HIF3A
axo18467-f	DUSP6
axo18474-f	HOXC8
axo18484-r	FANCF
axo18502-r	SMYD3
axo18525-f	ZNF574
axo18585-f	NAIP
axo18649-f	ADRB2
axo18705-r	METT10D
axo18720-f	C16orf68
axo18756-r	FSD1
axo18771-f	DSC2
axo18791-f	GCC1
axo18841-f	ARMC7
axo18911-f	GAL3ST4
axo18944-f	VPS37B
axo18975-f	SNIP1
axo18978-f	ZNF750
axo19033-f	ZBTB3
axo19057-f	PLBD1
axo19065-r	RPP21
axo19128-f	RMI1
axo19187-f	ZNF703
axo19200-f	KIAA1305

axo19257-f	COQ10B
axo19293-f	ITPKC
axo19297-f	PLEKHO2
axo19323-f	C6orf27
axo19439-r	NDEL1
axo19454-r	TRIM8
axo19468-r	ITM2C
axo19480-f	CCNL2
axo19483-f	TMEM49
axo19487-f	C1QTNF3
axo19488-r	PHACTR1
axo19553-f	EPPK1
axo19554-f	EPPK1
axo19555-f	EPPK1
axo19556-f	EPPK1
axo19582-f	THAP2
axo19651-f	ESYT3
axo19652-f	SYT16
axo19660-r	RSPH3
axo19711-f	B3GNT5
axo19785-f	ARID5B
axo19786-f	ARID5B
axo19814-f	FAM96A
axo19818-r	SGK196
axo19827-r	ZNRF1
axo19865-f	SLC25A33
axo19866-f	SLC25A33
axo19887-f	FBLIM1
axo19900-f	FBLIM1
axo19904-f	HVCN1
axo20006-r	TUBB6
axo20047-r	COX4I2
axo20049-f	RBBP6
axo20078-f	PPAPDC3
axo20142-r	PPP1R15B
axo20162-f	CORO6

axo20202-f	ZNF3
axo20215-f	RAB11FIP4
axo20228-f	TNFRSF6B
axo20242-f	SEC23B
axo20243-r	CEACAM1
axo20256-f	CSRNP1
axo20322-f	C5orf30
axo20333-r	BTBD6
axo20591-f	CD63
axo20613-f	ID2
axo20626-f	MYC
axo21092-f	GADD45B
axo21212-f	C8orf4
axo21228-f	NFKBIA
axo21314-f	KRTAP9-2
axo21474-f	RAB7B
axo21583-f	LOC100201082
axo21645-r	Unknown
axo21673-f	Plaur
axo21748-r	Unknown
axo21955-f	LAT2
axo22108-r	SPF1
axo22112-f	Unknown
axo22113-r	ORF2p
axo22179-f	CATHL3
axo22270-f	Unknown
axo22411-f	Unknown
axo22435-f	AVD
axo22477-r	Unknown
axo22590-f	LOC100182628
axo22833-f	SCYA107
axo22851-f	CCNI2
axo22852-r	Unknown
axo23008-f	LOC100145494
axo23062-r	Unknown
axo23084-f	LOC587493

axo23305-f	TCN1
axo23458-r	Unknown
axo23486-f	Unknown
axo23497-f	Unknown
axo23514-f	Pdcd1lg2
axo23748-f	LOC423629
axo23840-f	HAS1
axo24017-f	Unknown
axo24047-f	Prod 1
axo24065-f	LOC100224741
axo24073-f	PLAC8
axo24100-f	LRRC32
axo24112-r	Lynx1
axo24154-f	RGD1310061
axo24218-f	IRS4
axo24241-f	Unknown
axo24246-r	lrfn5
axo24254-r	BEST1
axo24256-f	LOC100229160
axo24325-r	LOC100127309
axo24368-f	Kctd14
axo24372-f	LOC100153971
axo24418-r	HNRPD
axo24475-f	CAPZB
axo24518-f	Unknown
axo24536-f	Unknown
axo24689-f	Unknown
axo24724-f	Unknown
axo24727-f	Unknown
axo24780-f	Unknown
axo24800-f	Unknown
axo24828-f	Unknown
axo24932-f	Unknown
axo24951-f	Unknown
axo24960-f	Unknown
axo24963-f	Unknown

axo25001-f	Unknown
axo25151-f	Unknown
axo25161-f	Unknown
axo25163-f	Unknown
axo25310-f	Unknown
axo25326-f	Unknown
axo25372-f	Unknown
axo25380-f	Unknown
axo25386-f	Unknown
axo25517-f	Unknown
axo25542-f	Unknown
axo25603-f	Unknown
axo25655-f	Unknown
axo25671-f	Unknown
axo25678-f	Unknown
axo25716-f	Unknown
axo25738-f	Unknown
axo25755-f	Unknown
axo25775-f	Unknown
axo25776-f	Unknown
axo25840-f	Unknown
axo25845-f	Unknown
axo25872-f	Unknown
axo25915-f	Unknown
axo25916-f	Unknown
axo25934-f	Unknown
axo25946-f	Unknown
axo25950-f	Unknown
axo25958-f	Unknown
axo26057-f	Unknown
axo26108-f	Unknown
axo26126-f	Unknown
axo26187-f	Unknown
axo26199-f	Unknown
axo26232-f	Unknown
axo26282-f	Unknown

axo26284-f	Unknown
axo26423-f	Unknown
axo26495-f	Unknown
axo26948-f	Unknown
axo26965-f	Unknown
axo27044-f	Unknown
axo27123-f	Unknown
axo27140-f	Unknown
axo27164-f	Unknown
axo27284-f	Unknown
axo27294-f	Unknown
axo27297-f	Unknown
axo27317-f	Unknown
axo27323-f	Unknown
axo27325-f	Unknown
axo27340-f	Unknown
axo27407-f	Unknown
axo27453-f	Unknown
axo27459-f	Unknown
axo27516-f	Unknown
axo27648-f	Unknown
axo27716-f	Unknown
axo27740-f	Unknown
axo27746-f	Unknown
axo27841-f	Unknown
axo27877-f	Unknown
axo27892-f	Unknown
axo27923-f	Unknown
axo27940-f	Unknown
axo28076-f	Unknown
axo28115-f	Unknown
axo28218-f	Unknown
axo28249-f	Unknown
axo28425-f	Unknown
axo28553-f	Unknown
axo28620-f	Unknown

axo28711-f	Unknown
axo28724-f	Unknown
axo28854-f	Unknown
axo28867-f	Unknown
axo28909-f	Unknown
axo28933-f	Unknown
axo28974-f	Unknown
axo29060-f	Unknown
axo29074-f	Unknown
axo29103-f	Unknown
axo29167-f	Unknown
axo29181-f	Unknown
axo29182-f	Unknown
axo29241-f	Unknown
axo29265-f	Unknown
axo29299-f	Unknown
axo29366-f	Unknown
axo29401-f	Unknown
axo29410-f	Unknown
axo29429-f	Unknown
axo29492-f	Unknown
axo29567-f	Unknown
axo29615-f	Unknown
axo29672-f	Unknown
axo29705-f	Unknown
axo29708-f	Unknown
axo29757-f	Unknown
axo29776-f	Unknown
axo29778-f	Unknown
axo29786-f	Unknown
axo29789-f	Unknown
axo29801-f	Unknown
axo29817-f	Unknown
axo29826-f	Unknown
axo29829-f	Unknown
axo29843-f	Unknown

axo29854-f	Unknown
axo29867-f	Unknown
axo29936-f	Unknown
axo29937-f	Unknown
axo30095-f	Unknown
axo30200-f	Unknown
axo30218-f	Unknown
axo30244-f	Unknown
axo30368-f	Unknown
axo30381-f	Unknown
axo30415-f	Unknown
axo30439-f	Unknown
axo30446-f	Unknown
axo30504-f	Unknown
axo30516-f	Unknown
axo30546-f	Unknown
axo30572-f	Unknown
axo30611-f	Unknown
axo30622-f	Unknown
axo30656-f	Unknown
axo30768-f	Unknown
axo30787-f	Unknown
axo30823-f	Unknown
axo30825-f	Unknown
axo30858-f	Unknown
axo30883-f	Unknown
axo30887-f	Unknown
axo30916-f	Unknown
axo30922-f	Unknown
axo30960-f	Unknown
axo31238-f	Unknown
axo31290-f	Unknown
axo31296-f	Unknown
axo31321-f	Unknown
axo31329-f	Unknown
axo31371-f	Unknown

axo31468-f	Unknown
axo31494-f	Unknown
axo31499-f	Unknown
axo31535-f	Unknown
axo31536-f	Unknown
axo31559-f	Unknown
axo31572-f	Unknown
axo31593-f	Unknown
axo31610-f	Unknown
axo31614-f	Unknown
axo31622-f	Unknown
axo31624-f	Unknown
axo31667-f	Unknown
axo31681-f	Unknown
axo31695-f	Unknown
axo31698-f	Unknown
axo31710-f	Unknown
axo31716-f	Unknown
axo31729-f	Unknown
