# Supplementary material for: Gene expression during the first 28 days of axolotl limb regeneration I: Experimental design and global analysis of gene expression
Source: Regeneration (Oxf). 2015 Jun 19;2(3):120–36. doi: 10.1002/reg2.37 (PMC4860271; doi:10.1002/reg2.37)
Supplement: Supplementary file 2 — Table S2. Significantly overrepresented gene ontology terms identified for intervals of time during limb regeneration. [file REG2-2-120-s002.pdf]

Supplemental Table 2. Significantly over-represented gene ontology terms identified for intervals of time during limb regeneration.

0-1 DPA

Category	Term	Count	Genes	Prob
GOTERM_BP_FAT	GO:0030574~collagen catabolic process	8	MMP10, PRTN3, MMP8, MMP19, MMP3, MMP13, MMP2, MMP1	0.0018827
GOTERM_BP_FAT	GO:0006928~cell motion	39	ZFAND5, CCK, PTGS2, ENPP2, TNFRSF12A, ARF6, ZEB2, CDH2, CAPZB, TGFB1, TPM3, NRCAM, CTTNBP2, BDNF, OVOL2, CXCR4, SFTPD, TEKT2, IL1B, B3GNT2, PPAP2A, NR2F2, THBS1, CEACAM1, SCG2, IL8, LMX1B, ARID5B, NR4A2, CCNL2, PLAUR, LSP1, NDEL1, ITGA6, ID1, BTG1, HBEGF, FOXC1, NGFR	0.00235216
GOTERM_BP_FAT	GO:0009719~response to endogenous stimulus	35	LDLR, PTGS2, RHOQ, FOXO1, BCL2L1, MMP3, VGF, TGFB1, CCNE1, CTTNBP2, FOS, GOT1, BCHE, HMOX1, IL1B, PCSK9, THBS1, RNF14, MAT2A, GATM, CRYAB, SOCS1, NR4A2, FBP1, RCAN1, NR4A3, MMP13, ADIPOQ, JUNB, DDIT3, LEP, SDC1, DUSP1, SLC25A36, VLDLR	0.00249722
GOTERM_BP_FAT	GO:0009991~response to extracellular stimulus	23	BMP2, CCK, GATM, PTGS2, HSD17B2, NR4A2, ADIPOQ, DDIT3, TGFB1, LEP, CCNE1, FOS, SSTR2, DUSP1, ITGA6, BCHE, HMOX1, LIPG, IL1B, PCSK9, HSPA5, KLF4, VLDLR	0.00768026
GOTERM_BP_FAT	GO:0042127~regulation of cell proliferation	46	PTGS2, AIF1, PRTN3, NFKBIA, FOXO1, BCL2L1, MXI1, SESN1, TGFB1, TIMP1, CTTNBP2, BDNF, OVOL2, CCL3L1, HMOX1, SERPINE1, SFTPD, IL1B, PPAP2A, THBS1, NRG1, FGFBP1, MYC, SCG2, ODC1, CDC6, BMP2, IL8, KLF10, GJB6, MYCN, PTHLH, ADRB2, SSTR2, ID2, EREG, BTG1, F3, BTG3, TGIF1, MDM2, HBEGF, ADAMTS1, NGFR, PRKRIR, KLF4	0.03651238
GOTERM_BP_FAT	GO:0006916~anti-apoptosis	21	TNFRSF6B, CRYAB, NFKBIA, FOXO1, BCL2L1, CITED2, BDNF, SQSTM1, HMOX1, F3, TNFAIP8, MPO, IL1B, NAIP, FOXC1, HSPA5, NGFR, NRG1, THBS1, MYC, GSTP1	0.01566257
GOTERM_BP_FAT	GO:0051252~regulation of RNA metabolic process	78	TCEANC, RBM15B, ZNF530, SNIP1, SPI1, FOXO1, RHOQ, ZEB2, TGFB1, YBX1, CITED2, FOS, CCNE1, HOXC8, ZNF182, ZNF300, HMOX1, LRRFIP1, NR2F2, NFIL3, MYC, ZNF180, ALX1, BATF3, EGR1, ZFP36, ARID5B, ZNF543, TLE3, ZNF792, SPEN, ECSIT, HMGA1, JUNB, DDIT3, ZNF3, MYCN, ADRB2, PRDM9, NAB1, TGIF1, MDM2, FOXC1, CREM, TFCP2L1, ZNF510, ZNF367, NFKBIA, ZFP36L2, TSC22D2, OVOL2, SQSTM1, XBP1, JUND, MYOG, ZNF268, VEZF1, RNF14, BMP2, NFE2, LMX1B, ZNF567, KLF10, LMCD1, NR4A2, NR4A1, NR4A3, ADNP2, SALL4, ID2, ID1, ETS2, CSRNP1, ZNF214, ZNF418, HIF3A, HIVEP2, KLF4	0.02475392
GOTERM_BP_FAT	GO:0051094~positive regulation of developmental process	22	BMP2, PDLIM7, TNFRSF12A, KLF10, PLXNB2, JUNB, TGFB1, LEP, NRCAM, CCNE1, BDNF, ADRB2, NDEL1, ID2, BTG1, F3, JUND, TGIF1, IL1B, NGFR, THBS1, NRG1	0.04305954
GOTERM_BP_FAT	GO:0031328~positive regulation of cellular biosynthetic process	40	SPI1, NFKBIA, RHOQ, FOXO1, TGFB1, CITED2, CCNE1, FOS, OVOL2, SQSTM1, HMOX1, IL1B, MYOG, NR2F2, THBS1, MYC, RNF14, ALX1, EGR1, BMP2, HSP90AA1, NFE2, LMX1B, NR4A2, NR4A1, GRHL3, NR4A3, HMGA1, JUNB, DDIT3, PTHLH, ADRB2, SALL4, EREG, ETS2, CSRNP1, FOXC1, AREG, KLF2, KLF4	0.04310444
GOTERM_BP_FAT	GO:0031667~response to nutrient levels	20	BMP2, CCK, GATM, PTGS2, HSD17B2, ADIPOQ, DDIT3, TGFB1, LEP, CCNE1, SSTR2, DUSP1, BCHE, HMOX1, LIPG, IL1B, PCSK9, HSPA5, KLF4, VLDLR	0.02720872

GOTERM_BP_FAT	GO:0001944~vasculature development	22	ZFAND5, IL8, TNFRSF12A, TIPARP, MMP19, FOXO1, CDH2, MMP2, JUNB, CITED2, EREG, CXCR4, ID1, HMOX1, IL1B, FOXC1, NR2F2, VEZF1, THBS1, CEACAM1, CYR61, SCG2	0.02727271
GOTERM_BP_FAT	GO:0042330~taxis	12	CXCL14, IL8, ENPP2, CXCR4, CMKLR1, CCL3L1, SFTPD, IL1B, CCNL2, CYR61, PLAUR, SCG2	0.03676292

2-3 DPA

Category	Term	Count	Genes	
GOTERM_BP_FAT	GO:0007049~cell cycle	56	KIF23, MAD1L1, MAEA, TUBB2B, PTTG1, CCNE2, KIF2C, MLL5, CDCA8, CUL5, MCM7, DDX11, INCENP, TARDBP, PSMD1, VPS4B, CCNA2, STAG2, CDC7, CDC6, RBL1, CDC5L, MCM3, ESCO2, VASH1, NCAPD3, MCM6, UHRF1, SASS6, MAD2L1, CKS1B, HAUS6, BLM, NEK3, LRRCC1, ANLN, CEP55, CCNG2, NIPBL, NCAPG2, PPP3CA, TFDP1, MSH6, MSH2, NASP, ATR, TET2, SMC2, CDC25A, SMC4, MAPK12, ZNF318, CHAF1A, CHAF1B, MYH10, UBE2E1	5.51E-07
GOTERM_BP_FAT	GO:0000279~M phase	27	KIF23, MAD1L1, HAUS6, TUBB2B, NEK3, LRRCC1, ANLN, CEP55, PTTG1, CCNG2, KIF2C, CDCA8, NIPBL, DDX11, NCAPG2, TARDBP, INCENP, CCNA2, STAG2, CDC6, MSH6, SMC2, NCAPD3, CDC25A, SMC4, MAD2L1, ZNF318	5.88E-04
GOTERM_BP_FAT	GO:0051276~chromosome organization	31	PTGES3, SMARCAD1, ENY2, BLM, CBX1, TRRAP, PTTG1, MLL5, CDCA8, NIPBL, DDX11, NCAPG2, MSH6, NFE2, HIST1H1B, MSH2, NASP, RBL1, SMC2, NCAPD3, SMC4, MAD2L1, BAZ1B, SMARCE1, BPTF, SMARCA5, CHAF1A, RBM14, SMARCA2, CHAF1B, UBE2E1	0.00200607
GOTERM_BP_FAT	GO:0006259~DNA metabolic process	37	PTGES3, BLM, POLA2, PTTG1, MCM10, DNASE1L3, TK1, CCNE2, MLL5, MCM7, POLE2, CTGF, ORC6L, FEN1, CDC7, MSH6, CDC6, RAD51AP1, MSH2, NASP, PPFIBP2, TOPBP1, ATR, MCM3, MCM4, ORC1L, MCM5, CDC25A, ESCO2, MCM6, UHRF1, RRM2, RRM1, PCNA, CHAF1A, RBM14, CHAF1B	1.15E-04

9-10 DPA

Category	Term	Count	Genes	
GOTERM_CC_FAT	GO:0031012~extracellular matrix	15	COL4A2, COL4A1, CILP, HSPG2, OLFML2A, LAMA2, LAMA4, COL6A3, COL6A2, COL6A1, RELN, COL29A1, LAMB1, LOXL1, DST	5.77E-06
GOTERM_CC_FAT	GO:0005576~extracellular region	25	MMRN1, BDNF, HTRA1, COL6A3, COL6A2, IL1B, COL6A1, PTX3, LAMB1, LOXL1, ADAM28, COL4A2, COL4A1, CILP, HSPG2, OLFML2A, LAMA2, PTHLH, PROM1, LAMA4, TNFSF10, CLEC3B, RELN, COL29A1, DST	1.95E-04
GOTERM_CC_FAT	GO:0005604~basement membrane	7	LAMA2, LAMA4, COL4A2, COL4A1, HSPG2, LAMB1, DST	5.37E-04

18-20 DPA

GOTERM_BP_FAT	GO:0051789~response to protein stimulus	9	EGR1, FOS, HSP90AA1, MAP1B, ITGA2, NR3C1, SETD2, VARS, CYR61	0.0019629

22-24 DPA

Category	Term	Count	Genes	
GOTERM_BP_FAT	GO:0008203~cholesterol metabolic process	10	LDLR, CYP51A1, SQLE, INSIG1, HMGCS1, FDPS, MVK, LSS, PMVK, FDFT1	7.38E-07
GOTERM_BP_FAT	GO:0008202~steroid metabolic process	12	LDLR, CYP51A1, SQLE, INSIG1, HMGCS1, FDPS, MVK, LSS, PMVK, SC5DL, SC4MOL,FDFT1	8.32E-07
