# Supplementary figures and images for: Gene expression during the first 28 days of axolotl limb regeneration I: Experimental design and global analysis of gene expression
Source: Regeneration (Oxf). 2015 Jun 19;2(3):120–36. doi: 10.1002/reg2.37 (PMC4860271; doi:10.1002/reg2.37)

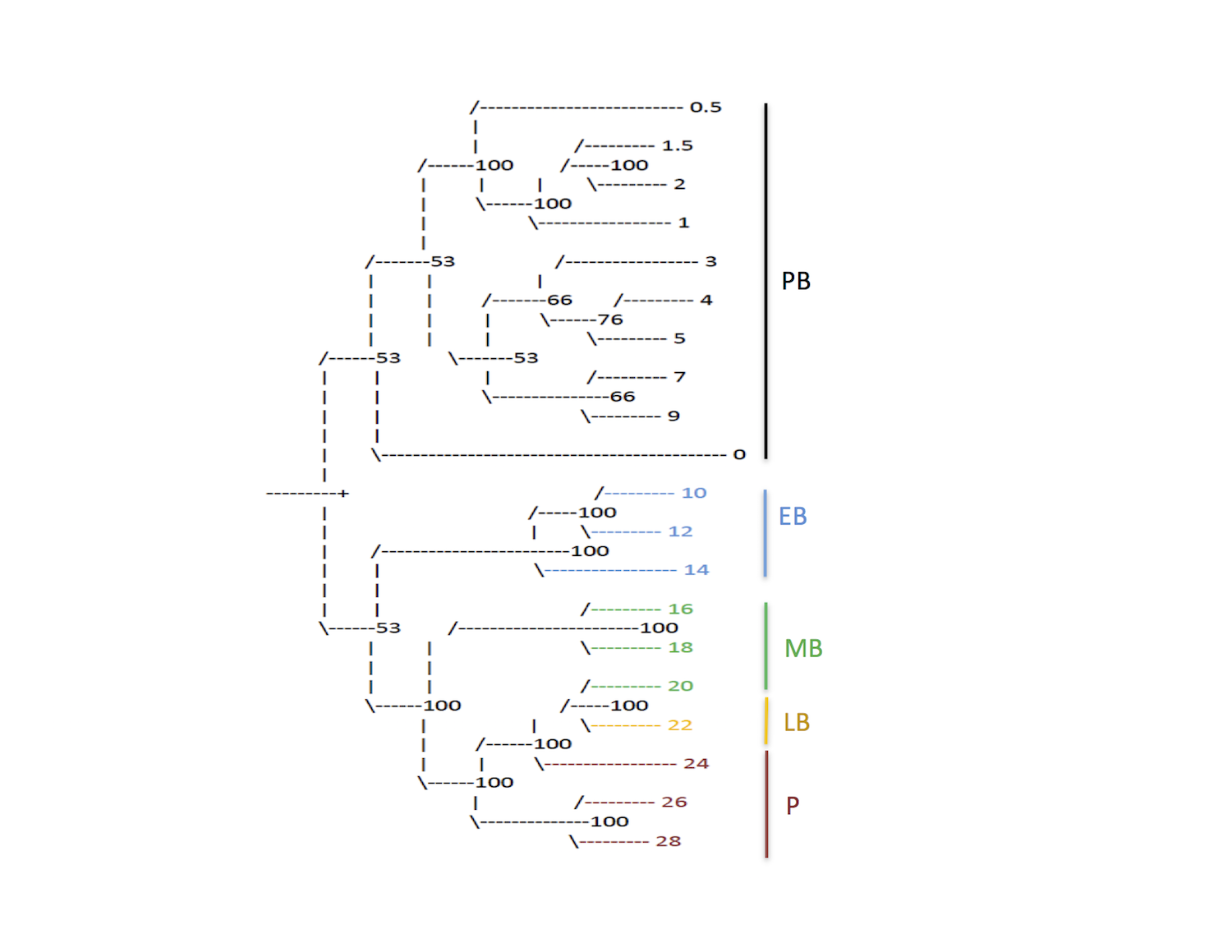

Supplement: Supplementary file 3 — Figure S1. Hierarchical cluster analysis reveals temporal grouping of samples, with the first bifurcation splitting samples into early (days 0−9) and late (days 10−28) groups. The numbers positioned at nodes in the dendogram are jackknifed probability values. The x‐axis shows the samples according to the day of collection. For example, 0, day 0; and 0.5, day 0.5. The colors refer to regeneration stages: pre‐bud (PB), early bud (EB), medium bud (MB), late bud (LB), and pallet (P). [file REG2-2-120-s003.tiff]
